# White matter alterations in crossing fibres following traumatic brain injury

**DOI:** 10.1093/braincomms/fcag042

**Published:** 2026-02-13

**Authors:** YiFan Jia, Niall J Bourke, Emma-Jane Mallas, Karen Caeyenberghs, Sara De Simoni, Peter O Jenkins, Juan Dominguez Duque, David J Sharp, Thomas D Parker

**Affiliations:** Department of Brain Sciences, Imperial College London, London, W12 0NN, UK; UK Dementia Research Institute Care, Research and Technology Centre, Imperial College London, London, W12 0BZ, UK; Department of Brain Sciences, Imperial College London, London, W12 0NN, UK; UK Dementia Research Institute Care, Research and Technology Centre, Imperial College London, London, W12 0BZ, UK; Kings College London, London, WC2R 2LS, UK; Department of Brain Sciences, Imperial College London, London, W12 0NN, UK; UK Dementia Research Institute Care, Research and Technology Centre, Imperial College London, London, W12 0BZ, UK; Cognitive Neuroscience Unit, School of Psychology, Deakin University, Burwood, VIC, VIC 3125, Australia; The Royal Hospital for Neuro-disability, London, SW15 3SW, UK; Division of Brain Sciences, Hammersmith Hospital, London, W12 0HS, UK; Department of Brain Sciences, Imperial College London, London, W12 0NN, UK; Hampshire Hospitals NHS Foundation Trust and University Hospital Southampton NHS Foundation Trust, Southampton, SO16 6YD, UK; Cognitive Neuroscience Unit, School of Psychology, Deakin University, Burwood, VIC, VIC 3125, Australia; Department of Brain Sciences, Imperial College London, London, W12 0NN, UK; UK Dementia Research Institute Care, Research and Technology Centre, Imperial College London, London, W12 0BZ, UK; Department of Brain Sciences, Imperial College London, London, W12 0NN, UK; UK Dementia Research Institute Care, Research and Technology Centre, Imperial College London, London, W12 0BZ, UK; Department of Neurodegenerative Disease, UCL Institute of Neurology, Queen Square, London, WC1N 3BG, UK

**Keywords:** fixel-based analysis, traumatic brain injury, diffusion MRI, diffusion tensor imaging, crossing fibres

## Abstract

Following traumatic brain injury, the ability of conventional diffusion-weighted MRI analysis techniques to resolve tract-specific white matter damage, particularly in crossing fibre regions, is limited. Using fixel-based analysis, this study aimed to identify white matter abnormalities in chronic traumatic brain injury patients and to resolve the effects of traumatic brain injury on distinct white matter tracts, especially in crossing fibre regions. In this cross-sectional study, diffusion-weighted MRI were acquired from adults with chronic moderate-to-severe traumatic brain injury (*N* = 29; median time since injury 1.9 years) and matched healthy controls (*N* = 17). Whole-brain and tract-of-interest analyses compared differences in white matter connectivity represented by fixel-wise metrics (fibre density, fibre bundle cross-section and combined fibre density and bundle cross-section) between groups. Regions where crossing white matter fibres demonstrates differential damage were identified. Significant reductions were found in all corrected fixel-wise metrics in traumatic brain injury patients, with distinct spatial distributions between metrics. Combined fibre density and bundle cross-section demonstrated the highest sensitivity out of the fixel-wise metrics and fractional anisotropy, detecting abnormalities in 73.6% of examined tracts. Fixel-based analysis resolved the distinct effects of traumatic brain injuries on crossing fibres with 14% of tract pairings containing crossing fibres (131/927) demonstrating robust evidence of differential damage (i.e. significant difference between groups in the fixel-wise metric of one tract in the pair but not the other tract within the same voxel). Fixel-based analysis identified variabilities in white matter abnormalities in traumatic brain injury patients. Crucially, fixel-based analysis was able to resolve injury-related tract-specific alterations even in crossing fibre regions, supporting further exploration of fixel-wise metrics as more specific biomarkers of white matter alterations in traumatic brain injury.

## Teaser

Jia *et al*. applied fixel-based analysis to patients with chronic traumatic brain injury, systematically revealing robust, differential damage in crossing white matter fibres missed by conventional methods. The authors demonstrate the technique’s potential as a more specific and interpretable biomarker for axonal injury.

## Graphical Abstract

**Graphical Abstract:**
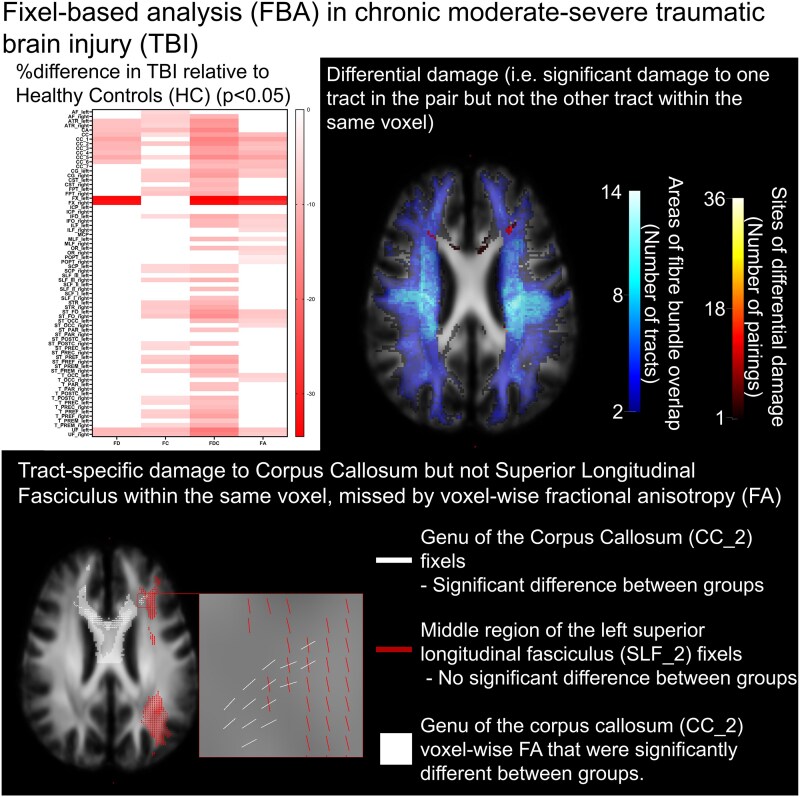

## Introduction

Traumatic axonal injury (TAI), caused by shearing forces to white matter (WM) axons, is a key component of traumatic brain injury (TBI) pathophysiology. This can be detected with diffusion-weighted magnetic resonance imaging (dwMRI), even in the absence of visible abnormalities on standard imaging sequences.^1^ Currently, diffusion tensor imaging (DTI) is the most widely used modelling technique applied to dwMRI. A tensor is fitted to each voxel to derive metrics of WM integrity such as fractional anisotropy (FA) and mean diffusivity (MD). DTI has been extensively used to examine changes post-TBI, with nearly all studies concluding widespread reductions in FA and increased MD, reflecting WM damage.^2-4^ Given the vulnerability of long-distance WM tracts to TAI, these changes have been consistently shown to be related to cognitive performance that relies on long-range pathways, such as executive function, processing speed and memory.^5^

Despite the significant insights DTI-derived voxel-wise metrics have provided, there are some inherent limitations that reduce their interpretability. As analysis is conducted at the level of a single tensor, in voxels with multiple fibre populations (i.e. crossing fibres), DTI is unable to model the effects of TBI on distinct WM tracts. In these regions, DTI metrics are averaged across all fibres in a voxel.^6,7^ Crossing fibres of this type are found in over 90% of WM voxels, making this a significant challenge for the assessment of TAI for a number of reasons.^8^ Firstly, damage to one tract may be balanced if other tracts in the voxel are undamaged, resulting in false-negative results. Secondly, damage to one tract whose orientation is perpendicular to the dominant tract within a voxel can counterintuitively result in increased FA.^9^ Finally, any damage detected cannot be attributed to specific tracts, which may limit its clinical applicability since damage to different tracts have different functional outcomes.^10^

Fixel-based analysis (FBA) is a novel DWI analysis technique that aims to address this limitation. Fixels are defined as distinct fibre populations within a voxel.^6,11^ By applying constrained spherical deconvolution to diffusion-weighted imaging data, FBA computes fibre orientation distributions (FODs) that represent the direction and properties of each fibre population in a voxel. This potentially increases its specificity to axonal properties, allowing damage to be detected in and attributed to specific WM tracts even in crossing fibre regions.^6^

**Figure 1:**
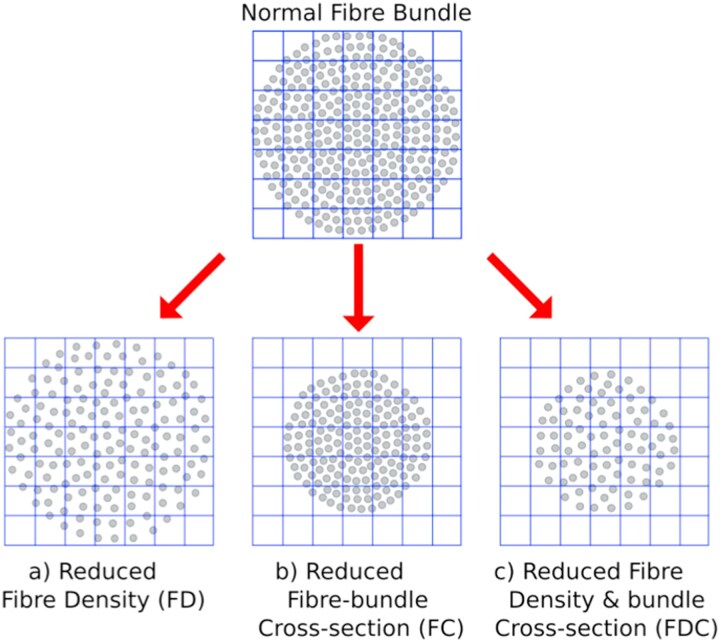
**Changes in different fixel-wise metrics.** The schematic represents a FC (grey circles represent individual axons, whilst boxes in the grid represent individual imaging voxels). Changes may be due to (**A**) altered within-voxel FD, (**B**) altered FC (**C**) alterations in a combination of FD and fibre cross-section. Figure republished from Raffelt *et al.*^6^ with permission of Neuroimage; permission conveyed through Copyright Clearance Centre, Inc.

This approach produces two complementary metrics: fibre density (FD), which aims to quantify changes in the fixel microstructure, defined as intra-axonal volume of a specific fibre population, and fibre bundle cross-section (FC), which estimates changes in fixel macrostructure, defined as morphological changes in the cross-sectional area of a fibre bundle. These metrics can be combined into a single measure of fibre density and cross-section (FDC) (Fig. 1).

Using FBA, previous studies have identified widespread WM alterations in both paediatric and adult TBI patient groups.^12-15^ However, its application in the chronic phase of TBI and in individuals with more severe injuries is limited. Importantly, whilst the ability of FBA to model crossing fibres is a known methodological advantage, the extent to which distinct tracts can be differentially vulnerable within the same voxel following TBI remains unknown.

We hypothesized that individuals in the chronic phase of moderate-to-severe TBI will have widespread alterations in WM connectivity, represented by significant decreases in fixel-wise metrics in patients relative to controls, and that these will correlate with markers of cognitive function. Furthermore, we aimed to investigate the prevalence and patterns of differential WM damage in crossing fibre regions in a chronic moderate-to-severe TBI cohort. We hypothesize that this differential injury is a frequent phenomenon, the characterization of which will help to improve the understanding of TAI pathophysiology.

## Materials and methods

### Participants

This study utilized an existing imaging dataset previously described.^16-18^ Participants included patients with moderate-to-severe (definite) TBI at least 3 months post-injury and age-matched healthy controls (HCs). Full entry criteria are provided in Supplementary Table 1. Significant inclusion criteria for both groups included (i) age between 20 and 65 years and (ii) provided written informed consent after their capacity was screened by a trained neurologist. Participants with histories of neurological or psychiatric illness, use of medications that may confound assessment, illicit drug use or contraindications to MRI scanning were excluded from this study. TBI was classified using the Mayo classification system,^19^ which considers moderate-to-severe (definite) TBI as the presence of one or more of loss of consciousness ≥ 30 min, post-traumatic amnesia > 24 h, lowest recorded Glasgow Coma Scale score < 13 in the first 24 h or abnormalities on standard neuroimaging (e.g. intracerebral/subdural/epidural haematoma, cerebral contusions, penetrating TBI, subarachnoid haemorrhage and brainstem injury). HC exhibited no clinically relevant impairments identified from detailed clinical history, physical examinations or clinical laboratory tests. All participants were prospectively recruited.

The final analysis cohort included 46 participants: 29 TBI patients and 17 HC (Supplementary Table 2). One patient and one control were excluded from this analysis cohort due to severe susceptibility distortions uncorrected by the pre-processing and an abnormally cropped field of view, respectively, both of which would have significantly biased the template mask if included. The study was approved by the West London and GTAC NRES Committee (14/LO/0067).

### Data acquisition

Imaging was acquired for all participants on the same Siemens Verio 3.0 Tesla scanner with a 32-channel head coil at the Imperial College Clinical Imaging Facility. Multi-shell DWI (30 gradient directions and b-value = 700 s/mm^2^; 60 gradient directions and b-value = 2000s/mm^2^) was performed with the following parameters: 66 contiguous slices, 128 × 128 acquisition matrix (2 mm^3^ isotropic voxels), and repetition time/echo time = 5000/105.2 ms. An additional nine images without diffusion weighting (b-value = 0 s/mm^2^) and a single reversed-phase encoding image without diffusion weighting were also obtained. The DWI acquisition time was 10 min. Furthermore, T1-weighted high-resolution magnetization prepared rapid acquisition gradient echo (T1 MPRAGE) was acquired for each participant with the following parameters: 160 1-mm-thick transverse slices, repetition time/echo time = 2300/2.98 ms, 1 mm^3^ isotropic voxel, flip angle = 9°, and 5 min scanning time.

All participants underwent a standardized neuropsychological battery to investigate common clinical dysfunctions after TBI (Table 1) as described in detail previously.^3^ Processing speed and executive function were assessed using the Trail Making Test (TMT), the Delis–Kaplan Executive Function System (D-KEFS) Colour-Word Interference Test (Stroop) and a choice reaction time (CRT) task;^20^ episodic memory was assessed using the Hopkins Verbal Learning Test – revised (HVLT-R), the Wechsler Memory Scale (WMS-III) logical memory subtests and the People Test (PT) from the Doors and People Test;^21-23^ reasoning ability and premorbid IQ were assessed using the Wechsler Abbreviated Scale for Intelligence (WASI) Matrix Reasoning and the Wechsler Test of Adult Reading (WTAR), respectively;^24,25^ finally, general psychological outcomes were assessed using behavioural reports, including the Lille Apathy Rating Scale (LARS), Hospital Anxiety and Depression Scale (HADS) and Short Form-36 (SF-36).^19,26,27^

**Table 1:** Clinical, demographic and neuropsychological assessment data of subjects

Domain	Characteristic	TBI	Control	*P*-value
Demographic	Age at visit (years)^a^	38.3 (1.9)	38.5 (2.6)	0.96
	Males^b^	25 (86.2)	14 (82.4)	0.31
Intracranial volume	Intracranial volume (mL)^a^	1457 (23.75)	1464 (31.90)	0.86
Injury characteristics	Time since injury (months)^c^	22.6 (104)	NA	NA
	Lowest GCS^c^	3 (5)	NA	NA
	Length of LOC (minutes)^c^	7.5 (15 800)	NA	NA
	Duration of PTA (days)^c^	21 (52)	NA	NA
Processing speed	Trail Making Test A (s)^c^	23 (24.5)	21 (11)	0.06
	Trail Making Test B (s)^c^	58 (42)	51.6 (20)	0.26
	Stroop Colour Naming and Word Reading Composite Score (s)^c^	30 (10)	25 (9.5)	0.06
	CRT Median RT (s)^c^	0.51 (0.11)	0.42 (0.08)	<0.01
Executive function	Trail Making Test B-A (s)^c^	32 (30.5)	33 (22.6)	0.96
	Stroop Inhibition (s)^c^	63 (21.5)	51 (18.5)	0.07
	Stroop Inhibition-Switching (s)^c^	73 (24)	55 (8)	<0.01
	Stroop Inhibition-Switching versus Baseline Contrast (s)^c^	41 (19.5)	32 (13.5)	<0.01
Memory	HVLT-R Immediate Recall^c^	23.5 (7.75)	27 (4.5)	0.04
	HVLT-R Delayed Recall^c^	9 (4.75)	10 (2)	0.04
	WMS-III Immediate Recall^c^	34 (15.5)	49.5 (10.3)	<0.01
	WMS-III Delayed Recall^c^	19 (14)	30 (13.8)	<0.01
	People Test Immediate Recall^c^	25 (10)	31 (9.5)	0.01
	People Test Delayed Recall^c^	8 (5.5)	12 (3)	0.09
Intellectual ability	WASI Matrix reasoning^c^	28 (5.5)	28 (7)	0.33
	WTAR Scaled^c^	110 (11)	119 (10)	0.01
Behavioural reports	LARS Self Total^c^	−24 (14.5)	−34 (4.25)	<0.01
	HADS-Anxiety^c^	7.5 (7.25)	5 (5.75)	0.03
	HADS-Ddepression^c^	7.5 (6.75)	3 (4.25)	<0.01
	SF-36 General Health^c^	60 (28.8)	77.5 (21.3)	<0.01
	SF-36 Physical Functioning^c^	80 (45)	100 (10)	<0.01
	SF-36 Emotional Wellbeing^c^	60 (23)	84 (17)	<0.01
	SF-36 Social Functioning^c^	62.5 (25)	100 (25)	<0.01
	SF-36 Energy/Fatigue^c^	35 (32.5)	67.5 (26.3)	<0.01

Unit used for the corresponding measurement, if applicable, is included in brackets.

Abbreviations: TBI, traumatic brain injury; NA, not applicable; GCS, Glasgow Coma Scale; LOC, loss of consciousness; PTA, post-traumatic amnesia; CRT, choice reaction time; RT, reaction time; HVLT-R, Hopkins Verbal Learning Test – revised; WMS-III, Wechsler Memory Scale; WASI, Wechsler Abbreviated Scale for Intelligence; WTAR, Wechsler Test for Adult Reading; LARS, Lille Apathy Rating Scale; HADS, Hospital Anxiety and Depression Rating Scale; SF-36, Short Form-36.

^a^Parametric data presented as mean (standard deviation). Reported *P*-value from unpaired, two-tailed *t*-test.

^b^Data presented as number (% of group). Reported *P*-value from chi-square test for independence.

^c^Non-parametric data presented as median (interquartile range). Reported *P*-value from unpaired, two-tailed Mann–Whitney U-test.

### Fixel-based pre-processing

**Figure 2:**
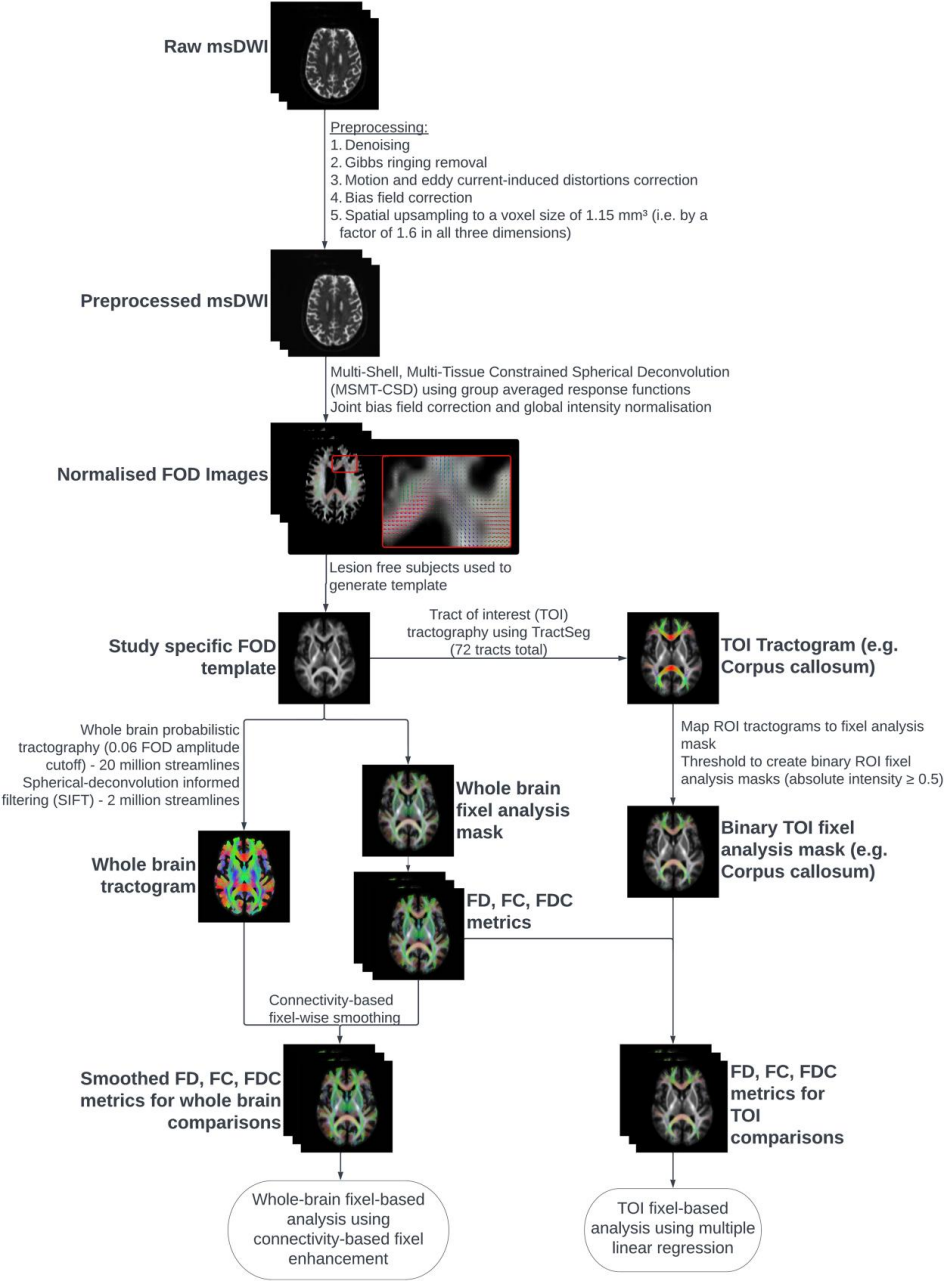
**Image processing and analysis pipeline for both whole-brain and tract-of-interest FBA using MRtrix3 and TractSeg.** ^28-30^ Lesion-free subjects (*n* = 23) were selected through visual inspection of T1-weighted images by two trained independent observers. msDWI, multi-shell DWI; FOD, fibre orientation distribution; FD, fibre density; FC, fibre bundle cross-section; FDC, fibre density and cross-section.

All DWI data were processed using MRtrix3 (version 3.0.3) following the official documented pipeline for multi-tissue constrained spherical deconvolution (Fig. 2).^28^ Visual quality control to check appropriateness of output was performed at each step of the processing pipeline.

In brief, initial pre-processing was performed, and the quality of dataset was assessed by two trained independent observers.^31-35^ FODs were computed using multi-shell, multi-tissue constrained spherical deconvolution using group averaged response functions computed from the whole cohort (*n* = 46) for WM, grey matter and corticospinal fluid.^6,36,37^ Joint bias field correction and global intensity normalization were then performed using these FODs.^38^

A study-specific FOD template was subsequently generated with an iterative registration and averaging approach using FOD images from 23 participants (7 TBI and 16 HCs) selected for their lack of significant lesions on visual inspection of T1-weighted images by two trained independent observers.^39^ FOD images from all participants were then registered and warped to the template space via FOD-guided non-linear registration to achieve spatial correspondence.^34,39-41^

Finally, whole-brain probabilistic tractography was performed on the FOD template,^39^ with the output filtered to two million streamlines to reduce reconstruction biases using spherical-deconvolution informed filtering of tractograms.^40^ Simultaneously, TractSeg was used to perform tract-of-interest tractography for each of the 72 documented tracts, producing 72 tractograms.^29,30^

### Voxel-based pre-processing

FA is currently the most common metric measuring WM integrity, so we additionally used MRtrix3 to perform voxel-based analysis.

For each subject, the diffusion tensor was estimated at each voxel of their pre-processed DWI using an iteratively re-weighted, linear least squares model, using the multi-shell diffusion data, and an FA map was generated by computing the FA of the diffusion tensors. Spatial correspondence was achieved by transforming these maps to the same population template space used in FBA.

### Total intracranial volume calculation

Total intracranial volume (TIV) was computed for each participant using a standard voxel-based morphometry pipeline on T1-weighted images using SPM12.^42^

### Lesion analysis

Focal lesion masks were generated for all patients and combined to form a lesion probability map in Montreal Neurological Institute (MNI) space (Supplementary Fig. 1).

### Statistical analysis

Demographic data and TIV were summarized as mean ± standard deviation (SD) and significant differences between groups tested using unpaired *t*-tests for age at visit and TIV and chi-square test for sex. All other data were summarized as median ± interquartile range (IQR) as they were non-parametrically distributed from visual inspection of histograms, and Mann–Whitney U-tests were used to test for significant differences between groups.

MRtrix3 was used for all whole-brain FBA (i.e. comparison of all WM fixels identified within the brain) and visualizations (Fig. 2).^28^ A whole-brain fixel analysis mask was first generated using the FOD template (threshold = 0.06 applied to the average FOD amplitude), and fixel-wise metrics were calculated for each masked fixel.^10^ Log_10_(FC) was calculated and used instead of FC for all whole-brain FBA to ensure data were zero centred and normally distributed. Connectivity-based smoothing and statistical inference were then performed using the filtered whole-brain tractogram with default smoothing parameters (smoothing = 10 mm full-width half-maximum; C = 0.5; E = 2; H = 3) using connectivity-based fixel enhancement (CFE).^11^

Finally, statistical comparisons of fixel-wise metrics at each identified fixel between groups were performed using a general linear model, with age, gender and intracranial volume included as nuisance covariates. Family-wise error (FWE)-corrected *P*-values were then assigned to each fixel using non-parametric permutation testing (5000 permutations).^43^

Tract-of-interest analysis was further performed to compare the degree of alteration between tracts and allow correlations with neuropsychological outcomes. Seventy-two tract-of-interest fixel analysis masks were generated using MRtrix3 and TractSeg from the tractograms,^28-30^ allowing mean metrics to be computed across the fixels in each defined tract for each participant (Fig. 2). FC was used for tract-of-interest analysis [rather than Log_10_(FC)], so percentage differences between groups are comparable between the three metrics, similar to approaches adopted in other studies.^9,12^

Multiple linear regression models with robust standard errors to account for heteroscedasticity were used to calculate the group mean difference in each metric for each tract following adjustment for age, sex and TIV false discovery rate (FDR) corrections of *P*-values for multiple group comparisons were then performed to similarly control for false positives.^44^

All tract-of-interest analyses were done in Python 3.7, with figures generated using GraphPad Prism 9.0.2.

Voxel-based smoothing, whole-brain analysis and tract-of-interest analysis were performed using the same method as in FBA, with the only differences being threshold-free cluster enhancement instead of CFE in whole-brain analysis (E = 0.5; H = 2) and voxel-analysis masks instead in tract-of-interest analysis.^45^

### Identifying crossing fibre dissociation

Crossing fibre dissociation voxels are defined as significant abnormalities in fixel-wise metrics of one tract, whilst one or more other tracts in the same voxel have no significant abnormalities.

The corpus callosum and superior longitudinal fasciculus were initially hypothesized as likely to have such dissociation voxels based on previous studies.^9^ To systematically identify other similar regions, all unique pairings of the 72 tracts that have crossing fibres were identified (927 out of 2556 unique pairings). For each of these pairings, the number of crossing fibre voxels showing dissociation for each metric was calculated.

For each metric, to ensure robustness of the results, pairings were filtered for significant dissociation according to the following criteria: (i) number of dissociation voxels > 10; (ii) dissociation voxels > 1% of total crossing fibre voxels; and (iii) dissociation at the whole tract level (i.e. only one tract in the pairing was significantly different between groups). The contiguity of dissociation voxels was also evaluated. There were at most two major clusters for each pairing, so this criterion was not used for further selection.

### Correlations with neuropsychological function

All whole-brain and tract-of-interest structural connectivity analyses were repeated in TBI patients to assess associations with selected neuropsychological outcomes with each WM integrity metric (FD, FC, FDC, or FA) as the independent variable. Results reflecting processing speed, memory, executive function and apathy were chosen as dependent variables based on previous literature.^46-48^ Age, gender and intracranial volume were included as nuisance covariates. Positive and negative contrasts were tested.

## Results

### Clinical, demographic and neuropsychological assessment data

Table 1 shows a summary of clinical, demographic and neuropsychological outcome data for the included participants (*n* = 29, 17; TBI, HC). There were no significant differences in age, sex or TIV between patients and controls. As expected, neuropsychological outcomes were significantly worse in the TBI group, particularly in executive function, memory and behavioural reports.

### Significant reductions in all fixel-wise metrics and voxel-wise FA were seen in the TBI group

**Figure 3:**
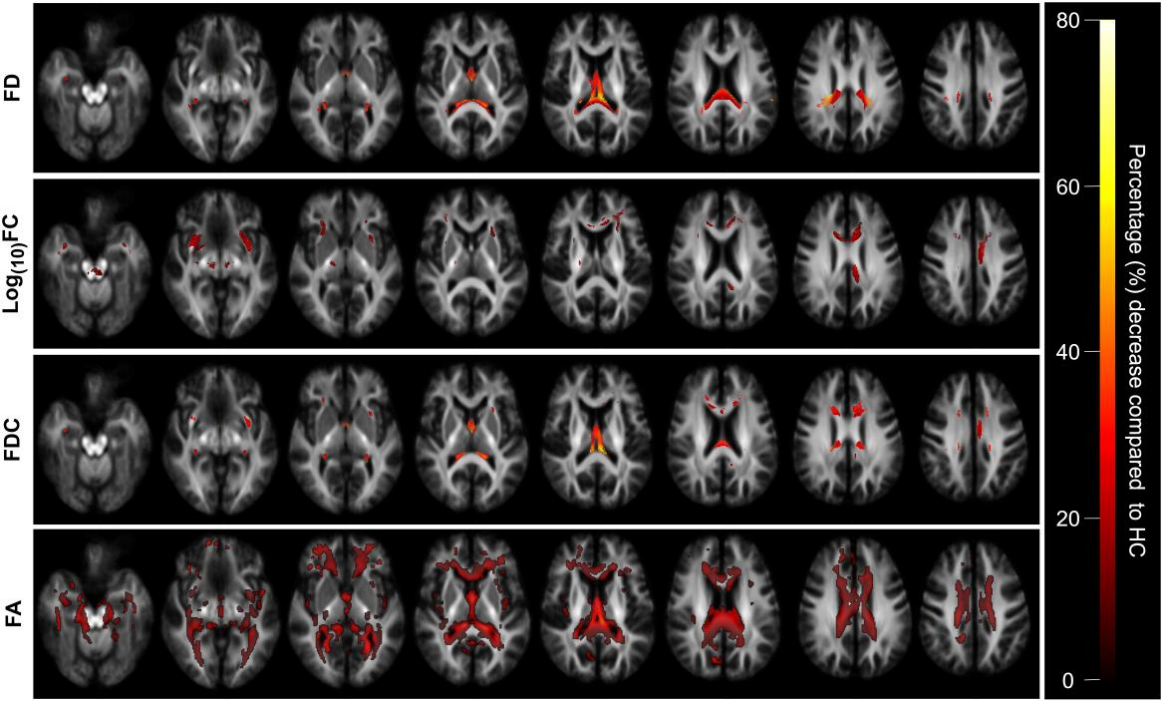
**Visualization of whole-brain fixel-based and voxel-based analysis showing reductions in all metrics in the TBI group.**  *Rows 1–3:* the whole-brain tractogram was thresholded to only show streamline segments corresponding to fixels that were significantly different (family-wise error-corrected *P* < 0.05) between groups after adjusting for nuisance covariates (age, sex and intracranial volume). Significant streamlines are displayed across eight axial slices of the study-specific FOD template. Streamlines were coloured by the percentage decrease in the TBI group relative to the HC group for each fixel-wise metric (FD, FC, and FDC). *Row 4:* voxels that were significantly different (family-wise error-corrected *P* < 0.05) between groups after adjusting for nuisance covariates (age, sex and intracranial volume) are shown across eight axial slices. These were coloured by the percentage decrease in the TBI group relative to the HC group with the same colour bar applied to both analyses.

Figure 3 shows thresholded streamline segments from the whole-brain tractogram corresponding to fixels with significant group differences in each metric, coloured by the percentage decrease in the TBI group. Significant decreases in FD were largely symmetrical between hemispheres, predominantly affecting the posterior brain. Specific affected tracts include the anterior thalamic radiations, anterior commissure, corpus callosum, fornix, and uncinate fasciculi. Significant decreases in Log_10_(FC) were less pronounced, though far more spatially extensive and differed between hemispheres. Whilst there were some spatial overlaps between the two metrics, long association fibres, most notably the cingulum, arcuate fasciculus and frontal–parietal tracts, only exhibited significant decreases in Log_10_(FC).

**Figure 4:**
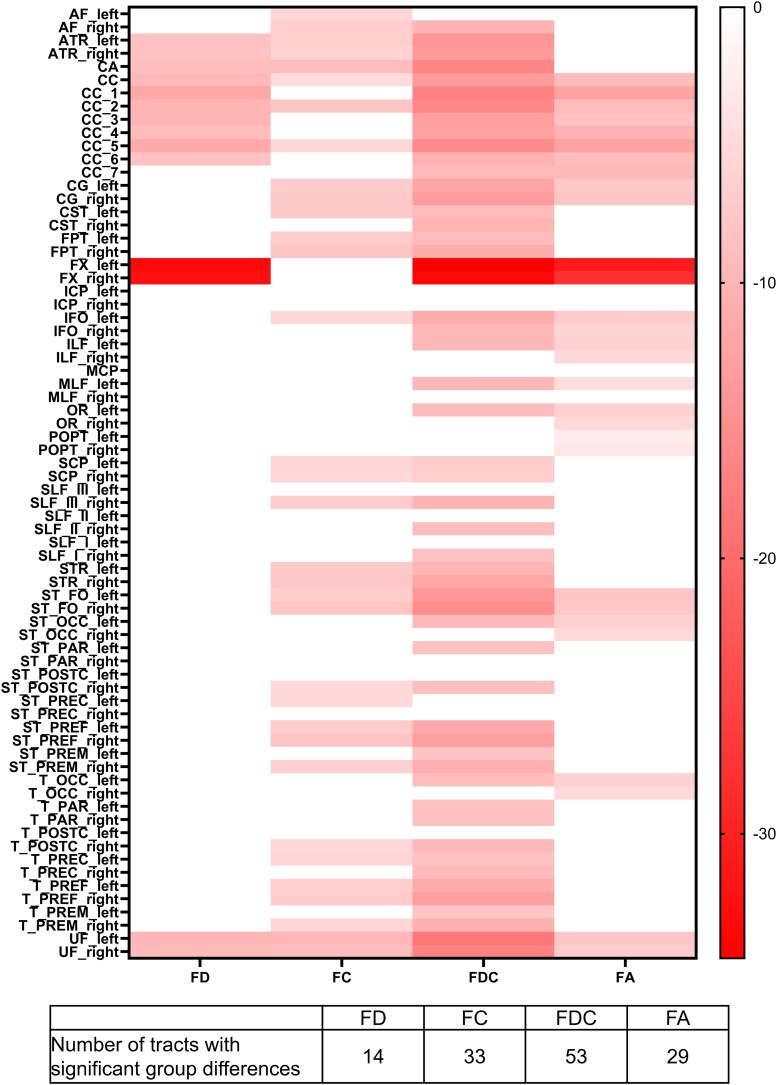
**Tract-of-interest analysis reveals reductions in all fixel-wise metrics and voxel-wise FA in the TBI group with similar patterns of tract involvement.** Heatmaps presenting the results of the multiple linear regression models to illustrate the group difference in mean fixel-wise metrics (FD, FC and FDC) and voxel-wise FA for each of the 72 tracts identified using TractSeg,^29,30^ coloured by the percentage change in the TBI group relative to the HC group. For each metric, only tracts with significant group differences (FDR-corrected *P* < 0.05) are coloured. Supplementary Tables 3–5 include additional summary information for all tracts. Mean metrics were calculated for each tract for each participant by averaging the metric’s value across all the fixels or voxels in the tract analysis mask. Multiple linear regression models were adjusted for nuisance covariates (age, sex and intracranial volume) and robust standard errors. FD, fibre density; FC, fibre bundle cross-section; FDC, fibre density and cross-section. The following list shows the full names of each tract: arcuate fascicle (AF), anterior thalamic radiation (ATR), commissure anterior (CA), corpus callosum [rostrum (CC 1), genu (CC 2), rostral body (CC 3), anterior midbody (CC 4), posterior midbody (CC 5), isthmus (CC 6), splenium (CC 7)], cingulum (CG), corticospinal tract (CST), middle longitudinal fascicle (MLF), fronto-pontine tract (FPT), fornix (FX), inferior cerebellar peduncle (ICP), inferior occipito-frontal fascicle (IFO), inferior longitudinal fascicle (ILF), middle cerebellar peduncle (MCP), optic radiation (OR), parieto-occipital pontine (POPT), superior cerebellar peduncle (SCP), superior longitudinal fascicle I (SLF I), superior longitudinal fascicle II (SLF II), superior longitudinal fascicle III (SLF III), superior thalamic radiation (STR), uncinate fascicle (UF), thalamo-prefrontal (T_PREF), thalamo-premotor (T_PREM), thalamo-precentral (T_PREC), thalamo-postcentral (T_POSTC), thalamo-parietal (T_PAR), thalamo-occipital (T_OCC), striato-fronto-orbital (ST_FO), striato-prefrontal (ST_PREF), striato-premotor (ST_PREM), striato-precentral (ST_PREC), striato-postcentral (ST_POSTC), striato-parietal (ST_PAR), striato-occipital (ST_OCC).

Similar patterns of tract involvement were shown in the tract-of-interest analysis. Figure 4 shows the group difference in mean fixel-wise metrics and for each tract, presented by percentage change in TBI relative to controls. Supplementary Tables 3–5 include additional summary information for all tracts. Far greater proportion of tracts exhibited significant decreases in mean FC and mean FDC in the TBI group than mean FD (19.4, 45.8 and 73.6% for FD, FC and FDC, respectively), and changes in mean FDC were additionally the most pronounced.

Voxel-based analysis revealed extensive significant FA reductions in TBI patients. Whilst significant FA changes appeared more widespread, the percentage differences were smaller compared to FDC (Fig. 3). In tract-of-interest analysis, FDC detected more tracts with significant differences between groups (Fig. 4). There were no significant increases in any metric in the TBI group in whole-brain or tract-of-interest analysis.

**Figure 5:**
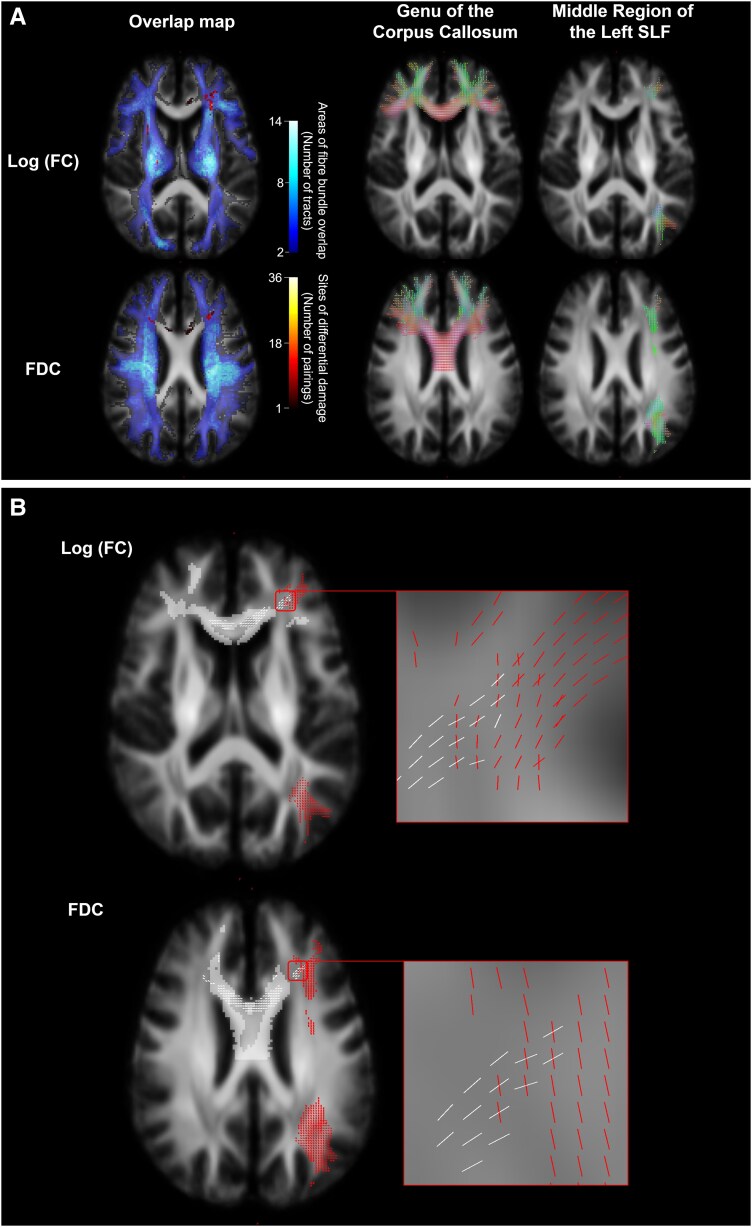
**Whole-brain visualization of voxels demonstrating differential damage in regions of crossing fibre.** (**A**) *Left:* WM voxels were coloured in blue based on the number of tracts present, only showing voxels containing fibre bundles from two or more tracts. For each of the pairings of tracts in these regions, voxels demonstrating differential damage (i.e. significant difference between groups in the FBA metric of one tract in the pair but not the other tract) are additionally overlayed. These were coloured in red/yellow, based on the number of such pairings per voxel. WM tracts were identified using TractSeg,^29,30^ excluding the subdivisions of the corpus callosum (*n* = 65). *Right:* Tract-of-interest fixel analysis masks with masked fixels coloured by direction (anterior–posterior, green; superior–inferior, blue; and left–right, red). Fixel analysis masks were produced using Mrtrix3 and TractSeg.^28-30^ (**B**) Fixels belonging to the genu of the corpus callosum that were significantly different (family-wise error-corrected *P* < 0.05) in the TBI group compared to HCs after adjusting for nuisance covariates (age, sex, and intracranial volume) for the Log_10_(FC) and FDC metrics are shown for a single axial slice of the study-specific FOD template in white. Red represents fixels belonging to the middle region of the left superior longitudinal fasciculus (SLF), none of which were significantly abnormal in these slices. The white squares represent voxels belonging to the genu of the corpus callosum that were significantly between groups. FD, fibre density; FC, fibre bundle cross-section; FDC, fibre density and cross-section.

Finally, two tracts were selected for additional graphical representation using FBA to highlight prominent sites of change in the TBI group (Fig. 5; Supplementary Fig. 2). These include the genu of the corpus callosum and the middle region of the left superior longitudinal fasciculus.

### Alterations in fixel-wise metrics are tract specific even in crossing fibre regions

Figure 5 highlights the tract specificity of fixel-wise metrics even in regions of crossing fibres. For example, fibres belonging to the corpus callosum and the superior longitudinal fasciculi cross extensively throughout the brain. In line with the a priori hypothesis and previous studies, FBA’s tract specificity allowed detection of decreases in Log_10_(FC) and FDC in fixels corresponding to the genu of the corpus callosum, without any significant abnormalities in fixels corresponding to the superior longitudinal fasciculus, despite them being in the same voxel (defined as crossing fibre dissociation voxels). Voxel-wise FA showed no significant abnormalities in these voxels (Fig. 5B). Fixels with a significant decrease in FD were not shown in this example as they did not cross with the left superior longitudinal fasciculus.

Such regions of crossing fibre were widespread. A total of 74.9% of WM voxels in the analysis mask contained fibre populations from two or more tracts. Systematic testing in these regions identified significant areas of differential damage in regions of crossing fibres (Fig. 5A; Supplementary Fig. 3).

Out of the 927 tract pairings that contain crossing fibres, 560 pairings showed differential damage in at least one voxel in one or more FBA metrics (60% of all pairings with crossing fibres). This includes 250, 350 and 235 tract pairings that showed differential damage for FD, FC and FDC, respectively (27, 38 and 25%, respectively).

A total of 131 of these pairings met all the defined criteria for significant dissociation (see Materials and methods) in one or more FBA metrics, including 78, 55 and 6 pairings for FD, FC and FDC respectively (Fig. 5; Supplementary Tables 6–8 and Fig. 3). It is important to note that there are few pairings for FDC due to the final criteria of requiring dissociation at the whole tract level between the tracts, as most tracts were significantly different between groups (67 pairings for FDC would meet both of the first two criteria).

### Associations of WM integrity metrics with neuropsychological function

Whole-brain analyses revealed significant positive correlations (FWE-corrected *P*-value < 0.05) between FD, FDC and FA with improved memory, measured by HVLT-R Delayed Recall, in TBI patients (Supplementary Fig. 4). This was most significant in the corpus callosum. Other positive correlations between WM integrity of tracts with measures of executive function, processing speed and apathy were also identified in tract-of-interest analysis, though these did survive multiple comparison correction (Supplementary Table 9).

## Discussion

Diagnosis and outcome prediction post-TBI remain a significant clinical challenge, so more sensitive and specific biomarkers are needed. This study showed significant decreases in all fixel-wise metrics and voxel-wise FA in the TBI group, identifying areas of disrupted WM connectivity. The spatial distribution of FD and FC changes was distinctive, suggesting TBI may have differential effects on WM tracts. Importantly, this systematic brain-wide investigation demonstrates that differential tract vulnerability in crossing fibre regions is a frequent phenomenon in TBI, with 14% of tract pairings containing crossing fibres (131/927) demonstrating robust evidence of differential damage.

To date, three other studies have applied FBA to a similar chronic moderate-to-severe TBI cohort.^12,14^ Generally, all studies revealed extensive reductions in all metrics, with the most consistent findings in major tracts, such as the corpus callosum and parts of the internal capsule. These FBA findings are supported by the significant reductions in voxel-wise FA in this study as well as the extensive DTI literature.^1,49^

However, there has been limited focus on crossing fibre regions. Given the direction-specific nature of shearing forces,^50^ TBI can even affect tracts within the same voxel differently. We use the well-established vulnerability of the corpus callosum as a primary example to demonstrate FBA’s ability to provide greater sensitivity than conventional voxel-based methods. Figure 5B demonstrates how fixel-wise FC and FDC showed tract-specific damage to the corpus callosum but not the superior longitudinal fascicle (SLF) within the same voxel, whereas voxel-wise FA showed no significant abnormalities in these voxels. Figure 5A demonstrates further widespread examples of differential tract damage in regions of crossing fibre. Fourteen per cent of tract pairings containing crossing fibres (131/927) demonstrated robust evidence of differential damage, highlighting the importance of tract specificity in the context of TBI.

This tract specificity may in part explain the improved sensitivity of fixel-wise metrics in detecting WM abnormalities compared to voxel-wise FA in this study and a previous DTI study on the same cohort.^10,16^ Whilst FA changes appeared more widespread in the whole-brain analysis, their effect sizes were smaller and became statistically insignificant when analysed at the individual tract level. In tract-of-interest analyses, only 40% (29/72) of analysed tracts showed significant FA differences between groups, compared to 73.6% (53/72) for FDC. Notably, many of these are long association fibres that cross extensively with other fibre pathways (Fig. 4). Voxel-wise FA is a normalized ratio influenced by alterations in extra-axonal signals—demyelination, inflammation, oedema and reactive gliosis, as well as changes (or lack thereof) in other tracts in the same voxel.^6,7^ Additionally, FA is more susceptible to partial volume effects. Mito *et al*.^9^ demonstrated how damage to one tract can even counterintuitively increase FA by reducing the tract’s relative contribution to the tensor, causing an increased anisotropy along the direction of other tracts in the voxel in the context of Alzheimer’s disease.

Such competing processes may also contribute to the distinct spatial distribution of FD and FC changes, highlighting the importance of having separate metrics for them (Figs 3 and 4) and may have several interpretations. Many tracts demonstrated significant decreases in FC with no change in FD in TBI patients. In chronic phases, though Wallerian degeneration driven by chronic neuro-inflammation causes progressive axonal loss, the demyelination and subsequent reactive gliosis, which causes dense glial scar formation and compaction of remaining tissue, results in macroscopic shrinkage of the entire fibre bundle. This results in fewer axons in a smaller cross-sectional area, manifesting as preserved FD with reduced FC. This would be consistent with previous findings of initial longitudinal studies showing decreases in FA followed by partial or complete recovery, whilst others exhibit persistent or even progressive damage.^15,51-53^ By providing more specific measures of axonal density and morphology, FBA metrics may help to disentangle these processes and provide insights into the long-term sequelae of TBI.

Interestingly, the fornix is a key structure that exhibited significant FD but not FC changes. This may indicate a milder or more acute form of injury than the presence of both, manifesting as axon loss only without significant atrophy of the fibre bundle. Technical factors may also contribute to these patterns. Periventricular structures such as the fornix are particularly vulnerable to partial volume effects from adjacent CSF. Our b-values (700 and 2000 s/mm²), whilst commonly used, may incompletely suppress extra-axonal signals. If the TBI group has greater ventricular volume (a common post-TBI finding), the fornix would experience greater CSF contamination, potentially causing FD reductions beyond true axonal density changes. Additionally, differences in fibre morphology of thin WM structures (within the scale of the voxel size), such as the fornix, are more likely to manifest as a change in FD instead of FC, owing to the effect of registration. Interestingly, the cingulum and anterior commissure—similarly thin periventricular structures—showed significant FC changes, suggesting that geometric factors alone cannot fully explain our findings.

Decreases in the FDC metric were both more spatially extensive and significant than in all other metrics, suggesting that it is the most sensitive metric to WM changes in TBI patients. This supports previous studies in other populations where FDC was found the most likely to reflect differences in WM connectivity and hence its ability to relay information, promoting the use of FDC as a diagnostic biomarker.^6,9^

Whilst whole-brain analysis revealed significant positive correlations between memory performance and WM integrity (FD, FDC, and FA), particularly within the corpus callosum, these associations did not survive FWE correction in tract-of-interest analyses. In addition to the statistical power being limited by a modest sample size and FWE corrections across 72 individual tracts, the heterogeneity of TBI populations further dampens potential findings. Our cohort, whilst representative of moderate-to-severe TBI, includes patients with diverse injury mechanisms, severities and time since injury, resulting in distinct patterns of damage between patients. This inherent limitation underlies the paradigm shift towards individualized analysis frameworks. Recent work by Jolly *et al*. (2021) and Clemente *et al*. (2023) demonstrates how individualized profiles can identify clinically relevant abnormalities missed by group-level analyses.^4,14^

As well as improving sensitivity and reliability of results, the advantages of FBA may aid clinical interpretability. The same abnormality in voxel-wise metrics may be linked to distinct clinical phenotypes depending on which specific tracts are affected. Similarly, the degree to which individual tracts are affected in terms of FD and FC may also influence its functional consequences. These may partly explain the contradictory findings of studies correlating voxel-wise metrics to clinical outcomes post-TBI.^49^ Given its ability to resolve distinct TBI effects on different tracts within the same voxel, FBA potentially could make more accurate discernments of the abnormalities’ causes and consequences.

Translating to clinical practice, this may help differentiate TBI into distinct pathological subtypes. Patients with isolated FD reductions might benefit from neuroprotective interventions targeting ongoing axonal loss, whilst combined FD/FC reductions may reflect established atrophy requiring different therapeutic strategies. This pathophysiological specificity may also provide endpoints for monitoring disease progression and treatment response. These potential clinical translations will require larger-scale longitudinal studies with more diverse TBI populations for robust validation.

In addition to being the first application of combined whole-brain and tract-of-interest FBA to a moderate-to-severe TBI cohort, this study also reports the first systematic evaluation of FBA’s ability to resolve tract-specific changes in crossing fibre regions. Its sample population ensured potential biases were minimized by controlling for confounders and using a clinically representative cohort.

However, this study has a few limitations. Firstly, whilst the DWI acquisition parameters were acceptable and in line with previous FBA papers, data suggests that the signal-to-noise ratio may be improved with ≥45 angular directions and b-values ≥ 3000 s/mm^2^.^10,54,55^ Additionally, the sensitivity of the FD metric is reduced at lower b-values due to an incomplete suppression of extra-cellular signals.^34,56^ Thus, actual changes in intra-axonal volume (represented by FD) may have been hidden by an increased extra-cellular volume, potentially contributing to fewer tracts having significant FD differences.

Whilst TractSeg demonstrates improved reproducibility and sensitivity, it has known limitations.^57,58^ The pre-defined 72 tracts exhibit significant overlap, so a single fixel may be assigned to multiple tracts, potentially inflating the number of distinct fibre populations identified in each voxel. The TractSeg atlas also does not provide 100% coverage of the WM, meaning our analysis is restricted to the pre-defined tracts.

Variabilities in the FBA pipeline due to user-defined parameters also has unknown consequences on the output. Whilst some default parameters exist, these are limited, and the extent to which they are generalizable beyond their initial experiments remains unknown.^11^ Similarly, the effect of injury characteristics (e.g. chronicity, mechanism and severity) on FBA processing steps and results remains relatively unexplored. Given the cross-sectional study design and modest sample size, this study was unable to investigate these. Furthermore, the FOD template generated from only seven TBI patients with minimal lesions, whilst necessary to avoid registration artefacts, may underrepresent inter-individual heterogeneity in the patient cohort. Many other pathological changes also occur simultaneously with WM damage post-TBI to which FBA is not sensitized, such as demyelination, grey matter atrophy and neuro-inflammation.^10,59^ Finally, data on whether individuals had undergone rehabilitation or received cognitive interventions were not available, though these may influence measures of WM integrity.

Future works should prioritize large-scale longitudinal designs in combination with improved acquisition parameters. Understanding how subject and injury characteristics impact FBA metrics may help resolve confounders between studies, especially given TBI’s heterogeneity. FBA metrics require histological validation and correlation with complementary imaging biomarkers (e.g. volumetric assessment, functional imaging and relaxometry) to establish their applicability in TBI.

In summary, this study demonstrated extensive significant tract-specific alterations in WM connectivity, largely consistent in pattern with previous literature. In addition, this study emphasized the importance of resolving differential damage in crossing fibre regions in the context of TBI. Through systematic brain-wide investigation, we demonstrate that differential tract vulnerability in crossing fibre regions is a widespread phenomenon in TBI, with 14% of tract pairings showing robust evidence of differential damage. Owing to their improved specificity and interpretability, future studies should aim to incorporate FBA in multimodal and/or longitudinal designs to thoroughly investigate the relationship between fixel-wise metrics, the underlying pathophysiological mechanisms and neuropsychological outcomes post-TBI. Ultimately, such an understanding would facilitate their use as accurate biomarkers, helping to tackle the significant clinical difficulty of predicting outcomes post-TBI, allowing for personalized interventions.

## Supplementary Material

fcag042_Supplementary_Data

## Data Availability

The data that support the findings of this study are available from the corresponding author, upon reasonable request.
