# Supplementary material for: White matter alterations in crossing fibres following traumatic brain injury
Source: Brain Commun. 2026 Feb 13;8(2):fcag042. doi: 10.1093/braincomms/fcag042 (PMC12960022; doi:10.1093/braincomms/fcag042)
Supplement: fcag042_Supplementary_Data [file fcag042_supplementary_data.docx]

# **Supplementary Table 1.** Inclusion and exclusion criteria

Inclusion criteria

1. A diagnosis of a moderate-severe traumatic brain injury (as defined by the Mayo TBI severity classification system) at least 3 months prior to recruitment into the study^1^

2. Age between 20 and 65 years

3. Capable of giving written informed consent

4. Subjective complaint of cognitive difficulties by the participant, treating clinician, or caregiver

Exclusion criteria

1. Unwillingness or inability to follow the procedures required

2. Significant neurological or psychiatric illness diagnosed prior to the TBI

3. Family history of a first degree relative with a psychotic illness

4. Currently participating in a clinical trial or has done so within 1 month before screening

5. Use of any medication or substance that, in the opinion of the investigators, would interfere with the study or compromise participant safety

6. History of a drug or other allergy that, in the opinion of the investigators, contraindicates their participation in the study

7. History of current or past drug or alcohol addiction

8. Female participants who are breast feeding or pregnant (positive pregnancy test) or plan to become pregnant during the study

9. Positive urine drug screen

10. Contraindication to MRI scanning, assessed by a standard pre-MRI questionnaire

11. Contraindication to the use of methylphenidate (including medications deemed to have a potentially serious interaction with methylphenidate as per the British National Formulary)

12. Clinical evidence of motor symptoms of Parkinsonism as assessed by a Neurologist

# **Supplementary Table 2 – Clinical demographics of individual participants**

| **Study ID** | **Group** | **Age** | **Gen der** | **Time since Injury (months)** | **Length of PTA (Days)** | **Cause of Injur y** | **Extracranial Injury Requiring Hospital** | **Days in Hospital** | **Site of impact** | **Lowest GCS** | **Length of LOC** | **Initial CT findings** | **MRI Findings** | **G O S E** | **U P D R S** | **Past Medical History** | **Medications at Visit** |
| --- | --- | --- | --- | --- | --- | --- | --- | --- | --- | --- | --- | --- | --- | --- | --- | --- | --- |
| DREAM016 | TBI | 4 6 | M | 37 | 15 | RTA | Yes | 14 | Frontal, Right | 14 | 1-29 minutes | SAH: Left Sylvian Fissure | Microbleeds: Parafalcine, Left Frontal, Right Frontal | 7 | 0 | Gastro- oesophageal reflux disease | None |
| DREAM017 | TBI | 2 0 | M | 23 | 60 | RTA | Yes | 50 | Occipital | 13 | 1-29 minutes | SAH; Fracture: Left Occipital Bone; Contusion: Left Frontal, Right Frontal, Left Temporal, Right Temporal, Left Parietal, Right Parietal; SDH: Right Parieto- Temporal | Contusion: Right Temporal Pole, Right Frontal Pole; Microhaemorrhage: Left Parietal Lobe, Left Frontal Lobe, Right Frontal Lobe, Left Temporal Lobe, Left Occipital Lobe | 5 | 0 | None | None |
| DREAM021 | TBI | 5 2 | M | 43 | 5 | RTA | Yes | 42 | Unknown | 15 | 0 - 1 Minute | Normal | Microhaemorrhage: Parafalcine, Left Frontal, Right Frontal | 6 | 0 | None | None |
| DREAM022 | TBI | 4 5 | M | 32 | 14 | Other Non-Intentional | No | 0 | Frontal, Occipital | 15 | 0 - 1 Minute | Not done | Normal | 6 | 0 | None | None |
| DREAM026 | TBI | 4 9 | M | 249 | 120 | Other Non Intentional | Yes | 120 | Frontal, Right | 3 | None | Unknown (in Bahamas) | Atrophy: Global, cerebellar, brain stem; Contusion: Right Frontal, Left Frontal, Right Temporal; Microhaemorrhage: Right Frontal, Left Frontal, Right Temporal | 5 | 3 | Rosacea | Sildenafil PRN, Loperamide PRN |
| DREAM028 | TBI | 2 4 | M | 8 | 14 | RTA | Yes | 15 | Right | Unknown | Unknown | Normal | Microhaemorrhage: Parafalcine, Right Frontal, Left Frontal, Left Parietal, Left lentiform nucleus, Left Temporal, Right Temporal | 6 | 0 | None | None |
| DREAM033 | TBI | 5 2 | M | 17 | 21 | Incident/ Fall | No | 85 | Occipital, Right | 8 | 1-29 minutes | SDH: Large Left with midline shift; SAH: Bilateral; Contusion: Left Frontal, Right Occipital; Fracture: Occipital bone, Sphenoid bone | Contusion: Left Temporal Lobe, Left Frontal Lobe, Right Frontal Lobe, Left Cerebellar Hemishere; Microhaemorrhage: Left Temporal, Left Frontal, Right Temporal, Right Frontal; Superficial Siderosis: Left Frontal, Left Temporal | 6 | 0 | None | Levetiracetam 1250mg BD, Perindopril 2mg OD |
| DREAM037 | TBI | 5 4 | M | 11 | 15 | Violence | No | 20 | Right | 12 | 1-24 Hours | SDH: Right; SAH: Left Frontal, Right Frontal; Contusion: Left Frontal, Left Temporal, Right Temporal, Right Occipital | Contusion: Right Temporal, Right Parietal, Right Frontal, Left Frontal, Left Temporal; Superficial Siderosis: Left Frontal | 5 | 0 | None | None |
| DREAM038 | TBI | 2 6 | M | 17 | 56 | Violence | No | 49 | Left, Occipital | Unknown | None | Contusion: Left Frontal, Left Temporal, Left Parietal; SAH: Left Parietal; SDH: Left | Contusion: Left Frontal, Left Parietal; Superficial Siderosis: Left Parietal, Left Frontal | 5 | 0 | None | Amitriptyline 10mg OD, Sodium Valproate 100mg OD |
| DREAM039 | TBI | 2 1 | F | 14 | 35 | RTA | Yes | 49 | Unknown | 14 | Unknown | Normal | Microhaemorrhage: Left Frontal, Corpus Callosum, Right Frontal | 6 | 0 | None | Fluoxetine 20mg OD, Propranolol 10mg PRN |
| DREAM040 | T B I | 3 9 | F | 6 | 2 | Incident/ Fall | No | 3 | Left, Frontal | 15 | 0 - 1 Minute | SDH: Tentorium cerebelli | Normal | 6 | 0 | None | None |
| DREAM041 | TBI | 5 4 | M | 24 | 1 | Incident/ Fall | No | 5 | Unknown | 10 | Unknown | Contusion: Left Temporal Lobe; SAH: Right Parietal, Left Temporal; Fracture: Left Squamous Temporal Bone | Contusion: Left Temporal Lobe; Microhaemorrhage: Right Temporal | 8 | 0 | Inflammatory Bowel Disease | Vitamin D |
| DREAM042 | TBI | 4 7 | M | 51 | 3 | RTA | No | 4 | Right, Occipital | 14 | 1-29 minutes | SAH: Bilateral; SDH: Right Frontal, Right Parietal, Left Frontal, Left Parietal; Fracture: Right Temporal Bone | Contusion: Right Parietal Lobe; Microhaemorrhage: Subcortical; Superficial Siderosis: Right Parietal Lobe, Left Frontal Lobe | 6 | 0 | None | Atorvastatin 10mg OD, Growth Hormone |
| DREAM044 | TBI | 2 2 | M | 21 | 540 | RTA | Yes | 90 | Crown | 3 | > 7 Days | SAH: Right Frontal, Left Frontal; Contusion: Splenium, Left Temporal | Contusion: Left Frontal, Right Frontal, Splenium, Left Thalamus, Left Temporal; Atrophy: Global; Microhaemorrhage: Subcortical, Parafalcine, Left Temporal, Right Temporal, Splenium, Right Frontal, Left Cerebellar | 4 | 3 3 | None | None |
| DREAM046 | TBI | 4 8 | M | 366 | 60 | RTA | Yes | 60 | Frontal | 3 | > 7 Days | Unknown | Contusion: Parafalcine, Left Frontal, Right Frontal; Microhaemorrhage: Left Temporal | 4 | 0 | Arthritis | Amitrptylline 20mg TDS, Esomeprazol e 40mg OD, Solifenacin 5mg TDS, Botox injections, Gabapentin 600mg TDS |
| DREAM047 | TBI | 3 8 | M | 210 | 720 | Violence | No | 180 | Unknown | 3 | > 7 Days | Unknown | Contusion: Left Occipital; Atrophy: Cerebellar, Pons, Left hemisphere | 5 | 7 | None | None |
| DREAM048 | TBI | 4 9 | F | 158 | 7 | Violence | No | 7 | Left, Occipital | Unknown | 1-29 minutes | Unknown | Normal | 5 | 0 | Hypothyroid | Levothyroxine 150 mcg OD, Omeprazole 20mg OD, Pyridoxine |
| DREAM052 | TBI | 3 6 | F | 219 | 120 | RTA | No | 160 | Frontal | Unknown | Unknown | Unknown | Contusion: Right Temporal Lobe; Microhaemorrhage: Right Frontal Lobe, Posterior Limb Right Internal Capsule, Right Occipital Lobe; Atrophy: Cerebellum, Pons | 4 | 4 3 | None | None |
| DREAM053 | TBI | 4 3 | M | 6 | 42 | RTA | Yes | 66 | Facial | 3 | 1-29 minutes | SDH: Right; Contusion: Right Frontal Lobe | Superficial Siderosis: Right Frontal Lobe (vertex); Microhaemorrhage: Right Frontal lobe, Corpus Callosum (Genu and Splenium), Mid Brain. | 5 | 0 | None | None |
| DREAM055 | TBI | 3 7 | M | 205 | 120 | Incident/ Fall | Yes | 150 | Right | 3 | > 7 Days | Unknown | Contusion: Right Frontal Lobe, Left Frontal Lobe, Left Temporal Lobe; Atrophy: Corpus Callosum, Left Temporal Lobe, Right Cerebral Peduncle; Previous Right Frontal Craniectomy | 5 | 0 | None | Tegretol CR 400mg BD |
| DREAM056 | TBI | 3 3 | M | 147 | 28 | RTA | No | 30 | Left, Occipital | 5 | Unknown | Fracture: Left Squamous Temporal Bone; Contusion: Right Frontal Lobe, Left Frontal Lobe, Left Temporal Lobe; SAH: Left Frontal, Left Temporal Lobes | Contusion: Right Frontal Lobe; Microhaemorrhage: Parafalcine, Right Frontal, Left Frontal; High Signal: Periventricular and Pontine, inkeeping with demyelination | 6 | 1 1 | None | Baclofen 20mg OD, Citalopram 10mg OD |
| DREAM057 | TBI | 3 4 | M | 9 | 7 | Incident/ Fall | No | 3 | Right, Occipital | 10 | 1-24 Hours | Normal | Contusion: Left Caudate Head; Microhaemorrhage: Left Temporal Pole, Left Occipital Lobe, Parafalcine (Left and Right Frontal Lobes) | 6 | 0 | None | None |
| DREAM060 | TBI | 3 2 | M | 73 | 28 | RTA | Yes | 28 | Unknown | 3 | > 7 Days | EDH: Right Temporal; Fracture: Right Temporal Bone, Right Greater Wing of Sphenoid, Left Frontal Bone, Left Greater Wing of Sphenoid, Left Maxillary Wall, Left Zygomatic Arch; SAH: Right Vertex | EDH: Left Frontal, Right Temporal, Left Occipital; SAH: Right Vertex; Contusion: Left Frontal Lobe; High Signal: Splenium; Microhaemorrhage: Left Frontal Lobe, Right Frontal Lobe | 6 | 0 | Asthma | Symbicort |
| DREAM061 | TBI | 5 2 | M | 23 | 4 | Violence | No | 3 | Occipital | 14 | Unknown | Fracture: Occipital bone | Contusion Right Frontal Lobe, Left Frontal Lobe, Left Temporal Lobe, Right Temporal Lobe | 6 | 0 | Gastro- oesophageal reflux disease | Lansoprazole 15 mg OD |
| DREAM063 | TBI | 3 8 | M | 9 | 14 | Violence | No | 14 | Occipital, Crown | 3 | 1-24 Hours | Contusion: Left Frontal Lobe, Right Frontal Lobe, Right Temporal Lobe; SDH: Parafalcine; SAH: Parafalcine; Fracture: Right Occipital Bone | Contusion: Right Frontal Lobe (superior frontal gyrus and frontal pole), Right Temporal Lobe (temporal pole), Left Frontal Lobe to a lesser degree; Superficial Siderosis: Vertex, right worse than left; Microhaemorrhages: Vertex, right worse than left | 6 | 0 | None | None |
| DREAM064 | TBI | 3 1 | M | 112 | 120 | RTA | No | 71 | Occipital | 6 | > 7 Days | Contusion: Left Frontal Lobe, Right Frontal Lobe, Corpus Callosum, Right Thalamus | Contusion: Parafalcine (Right and Left Frontal Lobes, worse on Right), Right Thalamus, Right and Left Parietal Lobes; Microhaemorrhage: Left Frontal Lobe, Right Frontal Lobe, Right Temporal Pole, Left Hippocampus; Atrophy: Cerebellum and globally | 5 | 1 1 | None | None |
| DREAM065 | TBI | 3 9 | M | 33 | 8 | RTA | Yes | 14 | Frontal, Right | 13 | 1-29 minutes | Contusion: Left Frontal Lobe | Contusion: Left Orbito- Frontal Lobe, Right Orbito- Frontal Lobe (worse on the left). | 5 | 0 | None | None |
| DREAM067 | TBI | 2 4 | F | 16 | 120 | RTA | No | 30 | Occipital, Left | 3 | Unknown | Contusion: Right Thalamus, Left Frontal Lobe, Right Frontal Lobe, Left Occipital Lobe, Right Occipital Lobe; SDH: Vertex | Contusion: Left Frontal Lobe, Right Frontal Lobe, Left Temporal Lobe, Corpus Callosum; Microhaemorrhage: Right Parietal Lobe, Left Temporal Lobe, Corpus Callosum; Atrophy: Splenium and Genu of Corpus Callosum | 6 | 1 | None | Citalopram 40mg OD |
| DREAM069 | TBI | 3 5 | M | 7 | 3 | Incident/ Fall | No | 5 | Left | Unknown | 1-29 minutes | Contusion: Right Frontal Lobe; EDH: Left; Fracture: Left Parietal Bone | Contusion: Right Frontal Lobe, Left Temporal Lobe, Right Temporal Lobe | 6 | 0 | Asthma | Salbutamol inhalers |
| DREAM024 | C o n t r o l | 4 3 | M |  |  |  |  |  |  |  |  |  |  |  |  |  |  |
| DREAM027 | C o n t r o l | 4 9 | M |  |  |  |  |  |  |  |  |  |  |  |  |  |  |
| DREAM029 | C o n t r o l | 4 4 | M |  |  |  |  |  |  |  |  |  |  |  |  |  |  |
| DREAM034 | C o n t r o l | 2 9 | M |  |  |  |  |  |  |  |  |  |  |  |  |  |  |
| DREAM035 | C o n t r o l | 3 4 | M |  |  |  |  |  |  |  |  |  |  |  |  |  |  |
| DREAM043 | C o n t r o l | 4 6 | M |  |  |  |  |  |  |  |  |  |  |  |  |  |  |
| DREAM049 | C o n t r o l | 4 7 | F |  |  |  |  |  |  |  |  |  |  |  |  |  |  |
| DREAM050 | C o n t r o l | 3 3 | M |  |  |  |  |  |  |  |  |  |  |  |  |  |  |
| DREAM051 | C o n t r o l | 2 5 | M |  |  |  |  |  |  |  |  |  |  |  |  |  |  |
| DREAM058 | C o n t r o l | 3 5 | M |  |  |  |  |  |  |  |  |  |  |  |  |  |  |
| DREAM059 | C o n t r o l | 2 4 | M |  |  |  |  |  |  |  |  |  |  |  |  |  |  |
| DREAM062 | C o n t r o l | 3 5 | M |  |  |  |  |  |  |  |  |  |  |  |  |  |  |
| DREAM068 | C o n t r o l | 5 7 | M |  |  |  |  |  |  |  |  |  |  |  |  |  |  |
| MEMCT01 | C o n t r o l | 2 8 | F |  |  |  |  |  |  |  |  |  |  |  |  |  |  |
| MEMCT02 | C o n t r o l | 3 5 | M |  |  |  |  |  |  |  |  |  |  |  |  |  |  |
| TauTBI_106 | C o n t r o l | 2 9 | F |  |  |  |  |  |  |  |  |  |  |  |  |  |  |
| TauTBI202 | C o n t r o l | 6 2 | M |  |  |  |  |  |  |  |  |  |  |  |  |  |  |

# **Supplementary figure 1 – Lesion probability map**


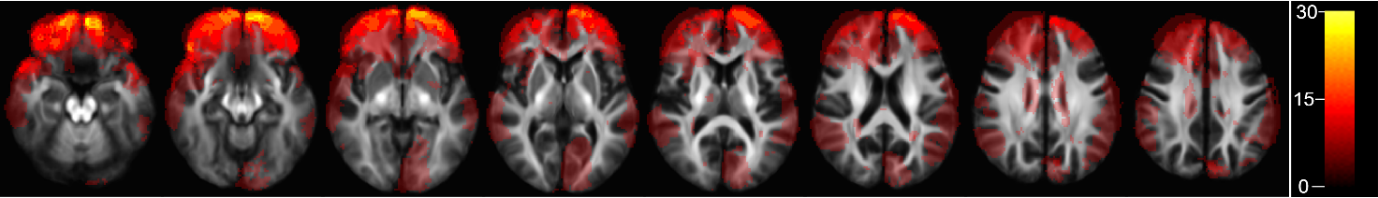


A binary lesion mask was manually delineated for each TBI subject for areas of contusion/missing brain. These binary masks were averaged across all patients to demonstrate areas of high overlap (bright white) and areas of low overlap (dark red).

# **Supplementary Table 3.** Differences in group mean fibre density (FD) for all 72 white-matter tracts between TBI patients (N=29) and healthy controls (N=17).


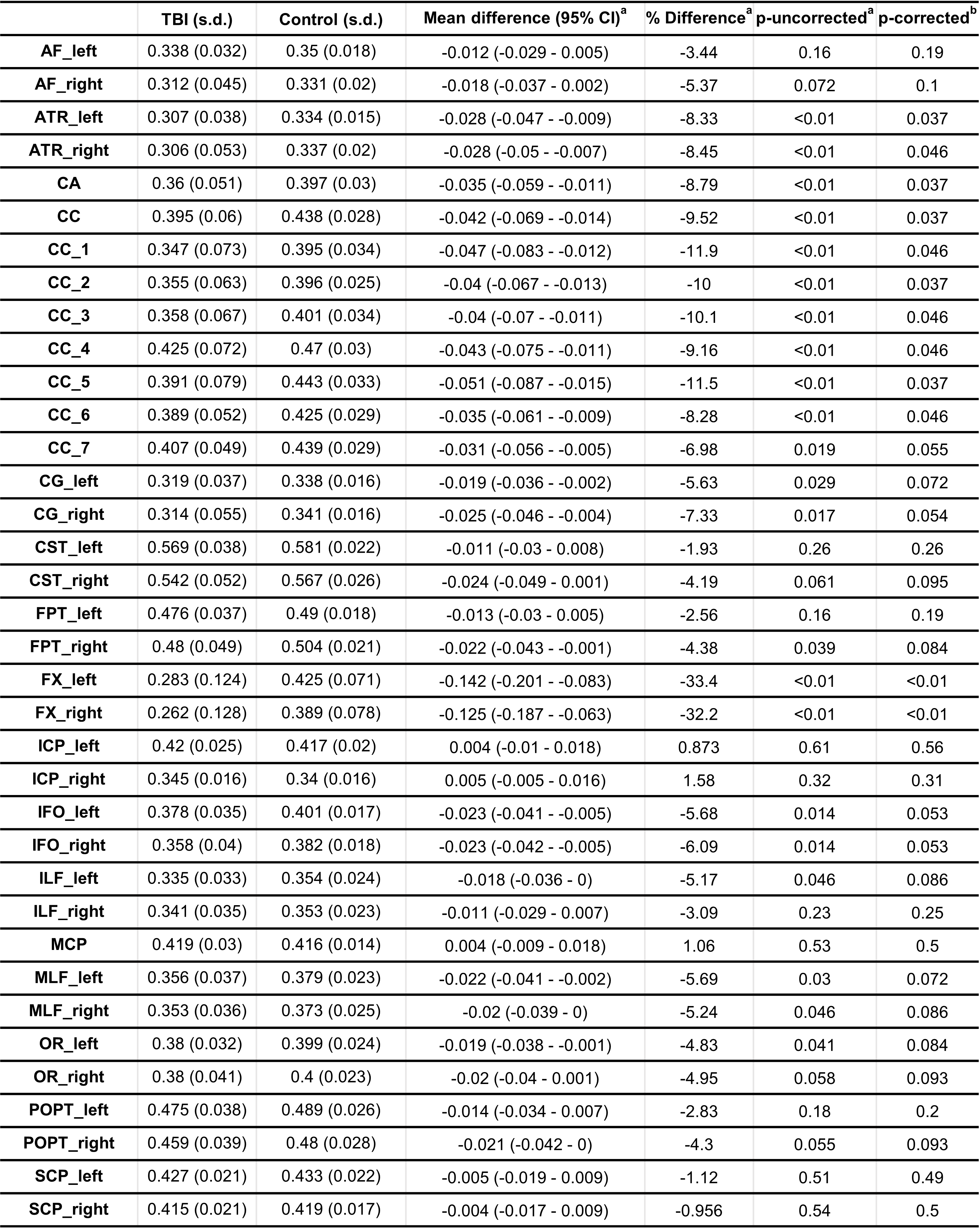


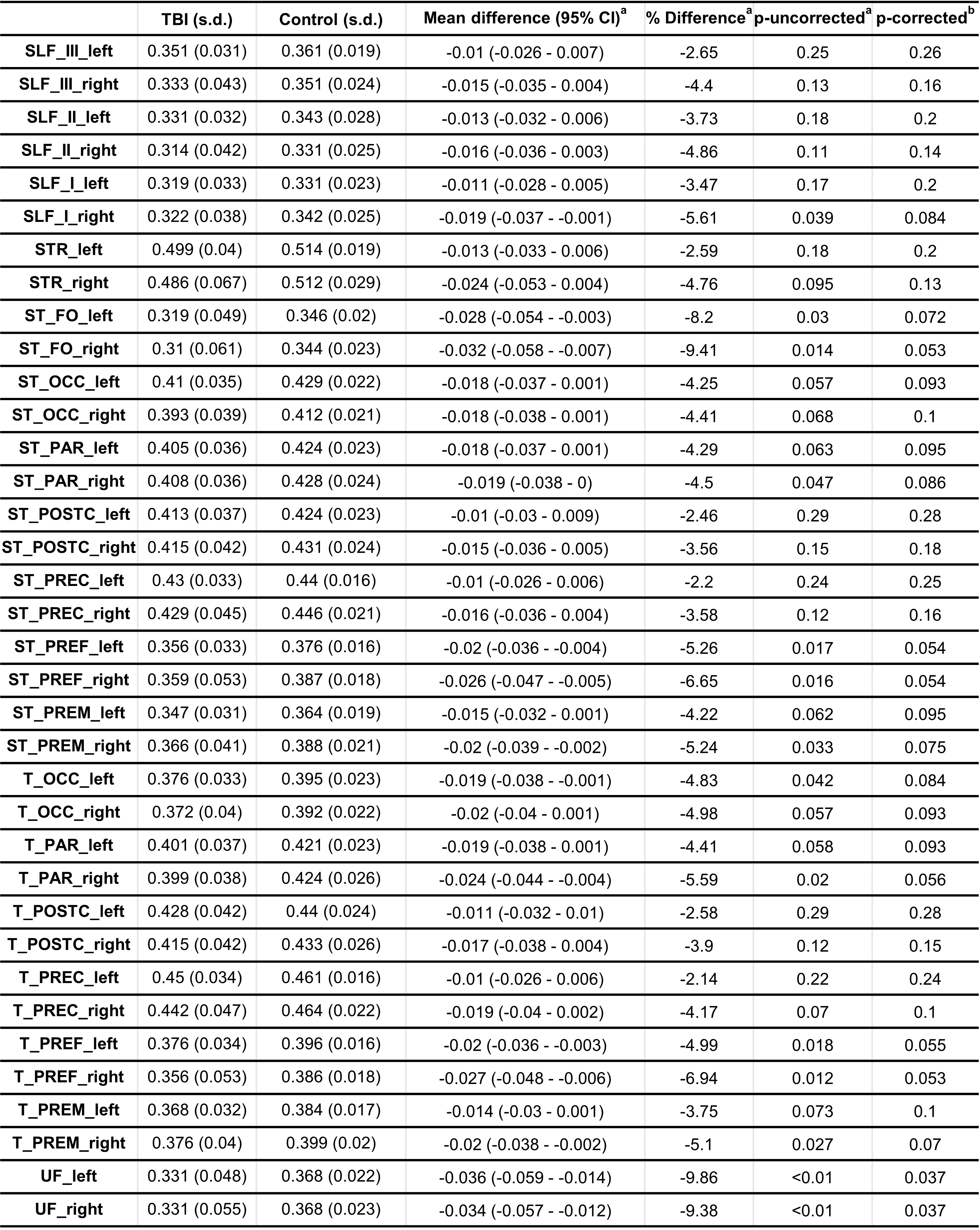


The full name of each tract can be seen in Fig. 4. Summary data are mean ± standard deviation. Group differences presented as the mean and percentage (%) difference of the TBI group relative to the control group.

^a^The mean differences (95% CI), % differences, and uncorrected p-values were obtained using multiple linear regression adjusted for nuisance covariates (age, sex, and intracranial volume) and robust standard errors.

^b^P-values were corrected for multiple comparisons via false-discovery rate correction (FDR) using the two-stage linear step-up method of Benjamini, Krieger and Yekutieli.^2^

Abbreviations used: TBI = traumatic brain injury; s.d. = standard deviation; CI = confidence interval.

# **Supplementary Table 4.** Differences in group mean fibre-bundle cross-section (FC) for all 72 white-matter tracts between TBI patients (N=29) and healthy controls (N=17).


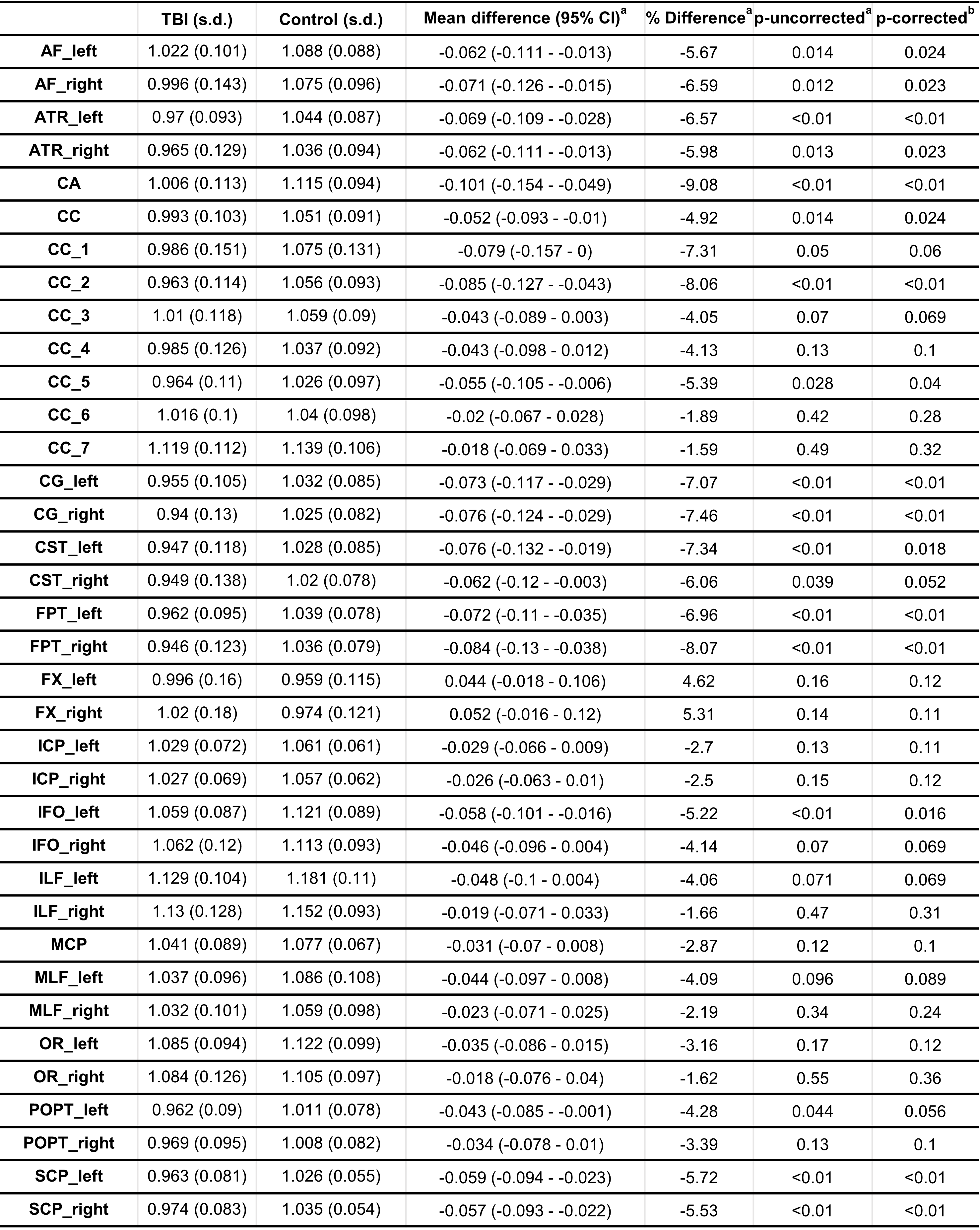


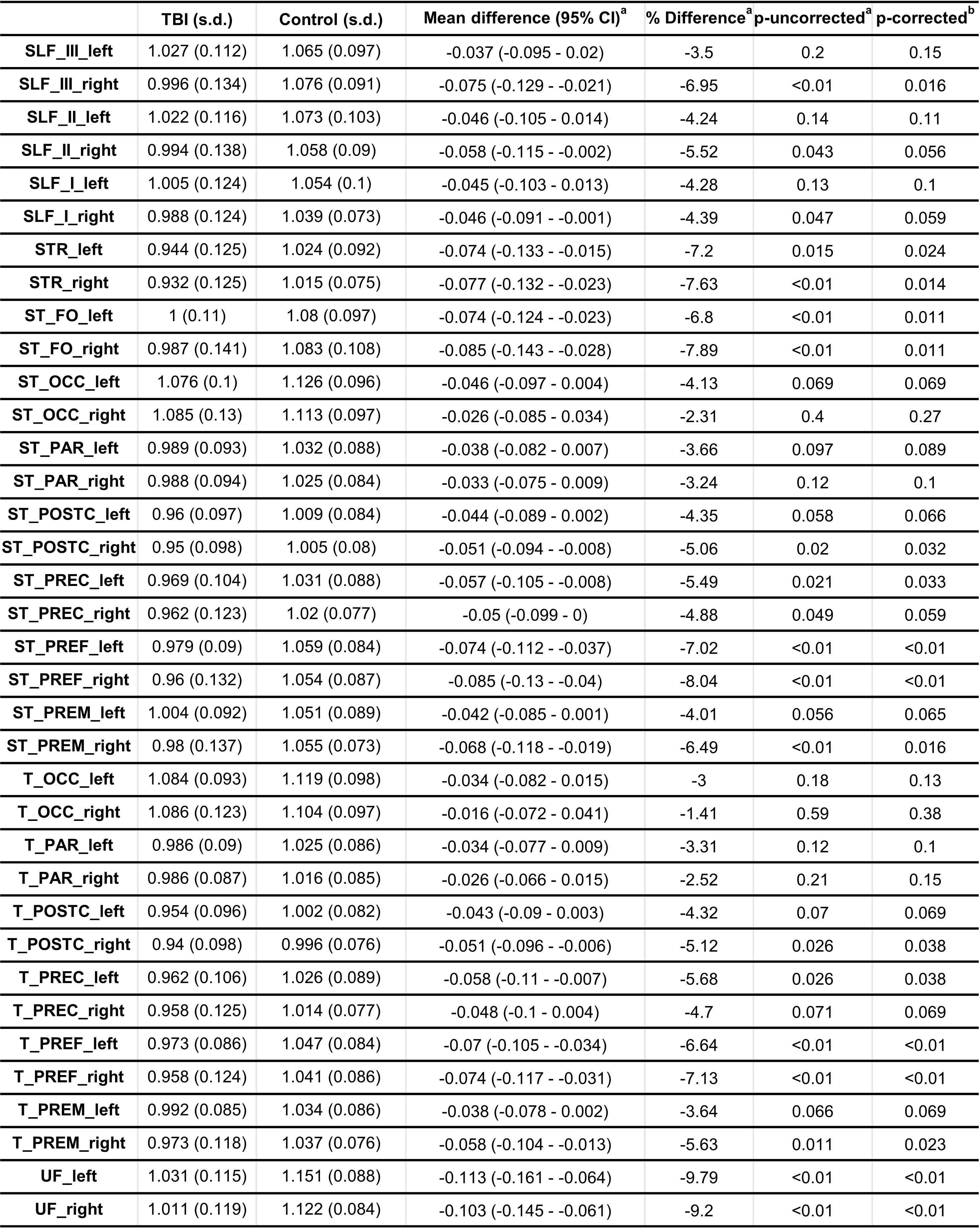


The full name of each tract can be seen in Fig. 4. Summary data are mean ± standard deviation. Group differences presented as the mean and percentage (%) difference of the TBI group relative to the control group.

^a^The mean differences (95% CI), % differences, and uncorrected p-values were obtained using multiple linear regression adjusted for nuisance covariates (age, sex, and intracranial volume) and robust standard errors.

^b^P-values were corrected for multiple comparisons via false-discovery rate correction (FDR) using the two-stage linear step-up method of Benjamini, Krieger and Yekutieli.^2^

Abbreviations used: TBI = traumatic brain injury; s.d. = standard deviation; CI = confidence interval.

# **Supplementary Table 5.** Differences in group mean fibre density & fibre-bundle cross-section (FDC) for all 72 white-matter tracts between TBI patients (N=29) and healthy controls (N=17).
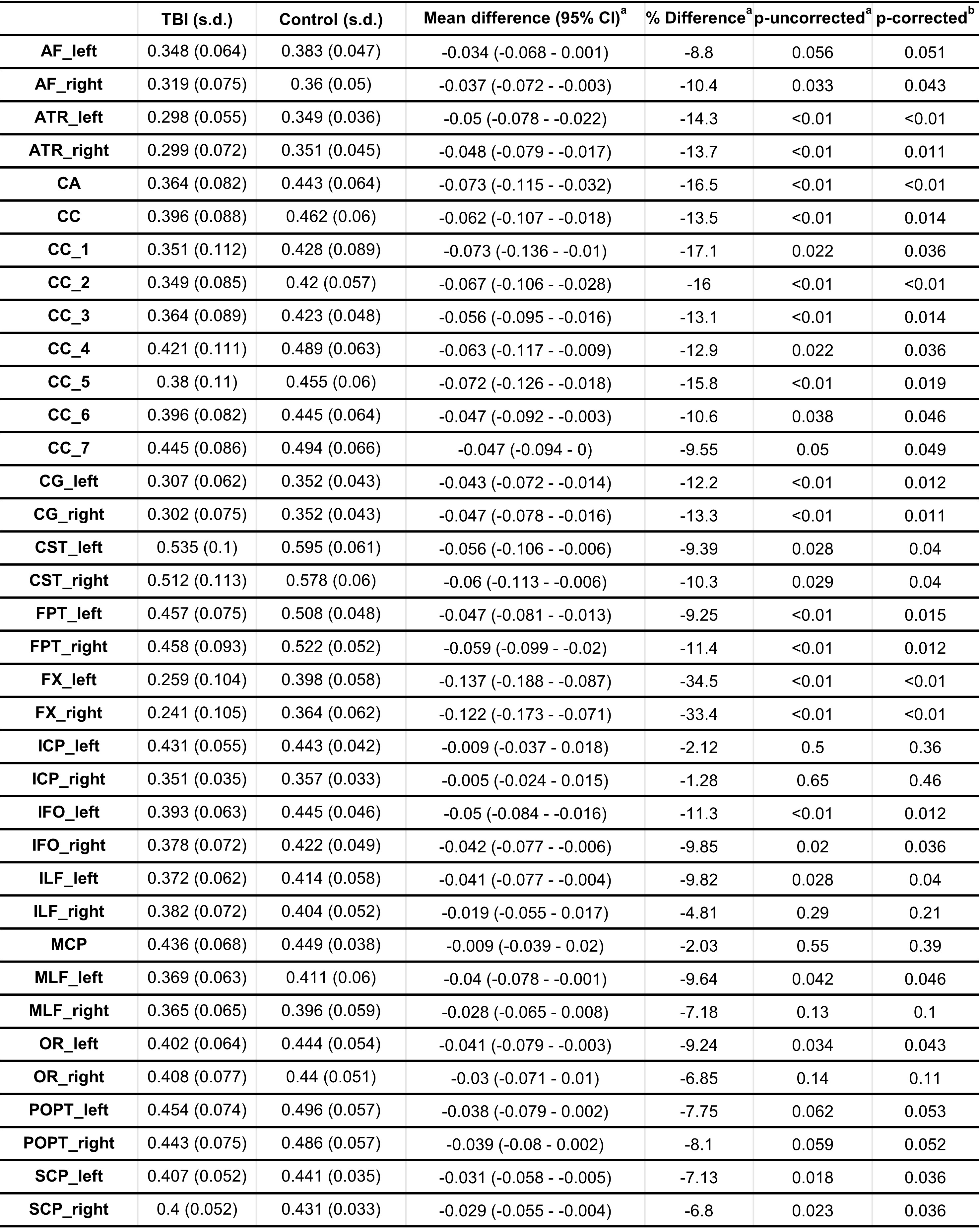


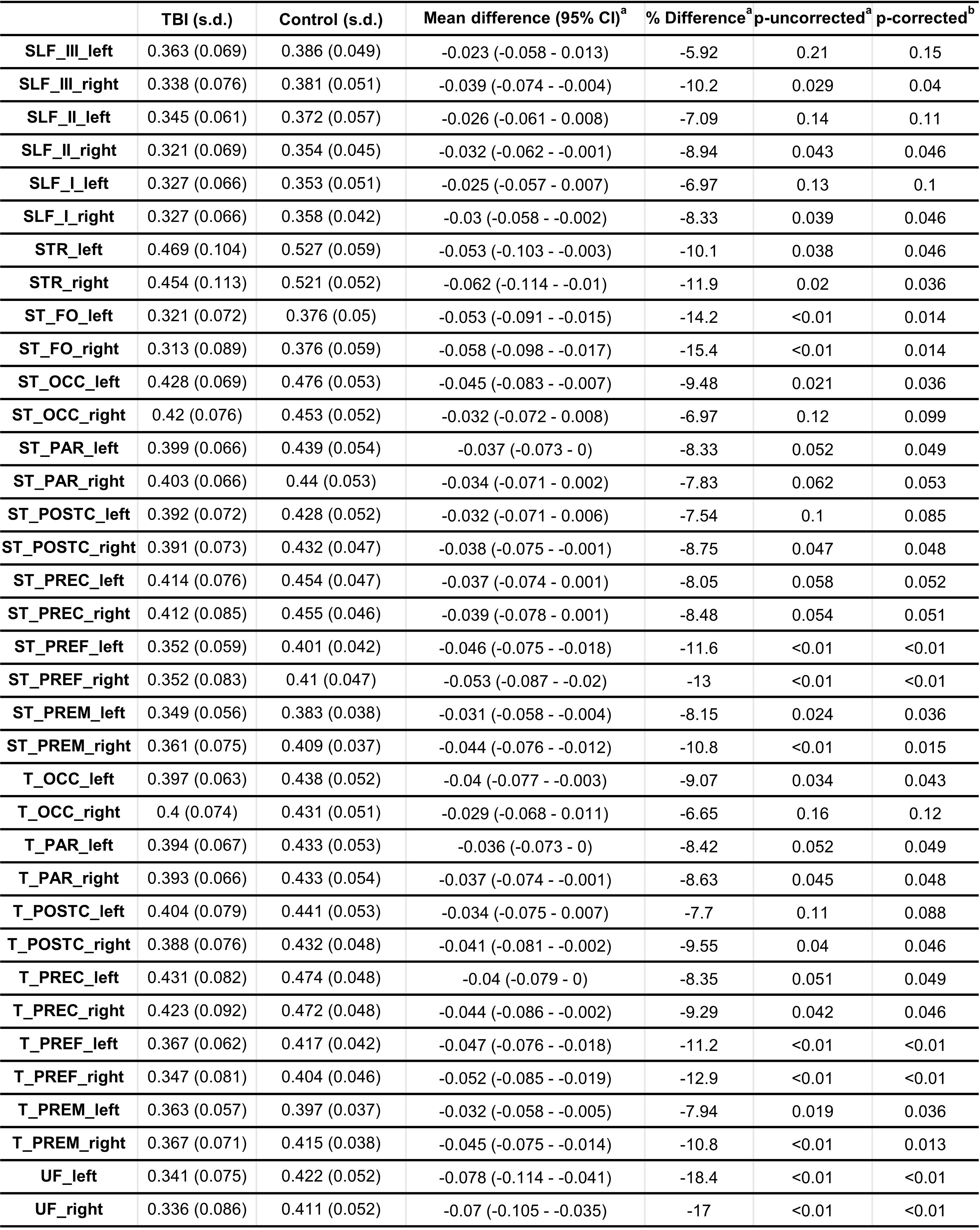


The full name of each tract can be seen in Fig. 4. Summary data are mean ± standard deviation. Group differences presented as the mean and percentage (%) difference of the TBI group relative to the control group.

^a^The mean differences (95% CI), % differences, and uncorrected p-values were obtained using multiple linear regression adjusted for nuisance covariates (age, sex, and intracranial volume) and robust standard errors.

^b^P-values were corrected for multiple comparisons via false-discovery rate correction (FDR) using the two-stage linear step-up method of Benjamini, Krieger and Yekutieli.^2^

Abbreviations used: TBI = traumatic brain injury; s.d. = standard deviation; CI = confidence interval.

3. Summary data are mean ± standard deviation. Group differences presented as the mean and percentage (%) difference of the TBI group relative to the control group.

^a^The mean differences (95% CI), % differences, and uncorrected p-values were obtained using multiple linear regression adjusted for nuisance covariates (age, sex, and intracranial volume) and robust standard errors.

^b^P-values were corrected for multiple comparisons via false-discovery rate correction (FDR) using the two-stage linear step-up method of Benjamini, Krieger and Yekutieli.^2^

Abbreviations used: TBI = traumatic brain injury; s.d. = standard deviation; CI = confidence interval.

# Supplementary Table 6 – FD filtered crossing fibre tract pairings


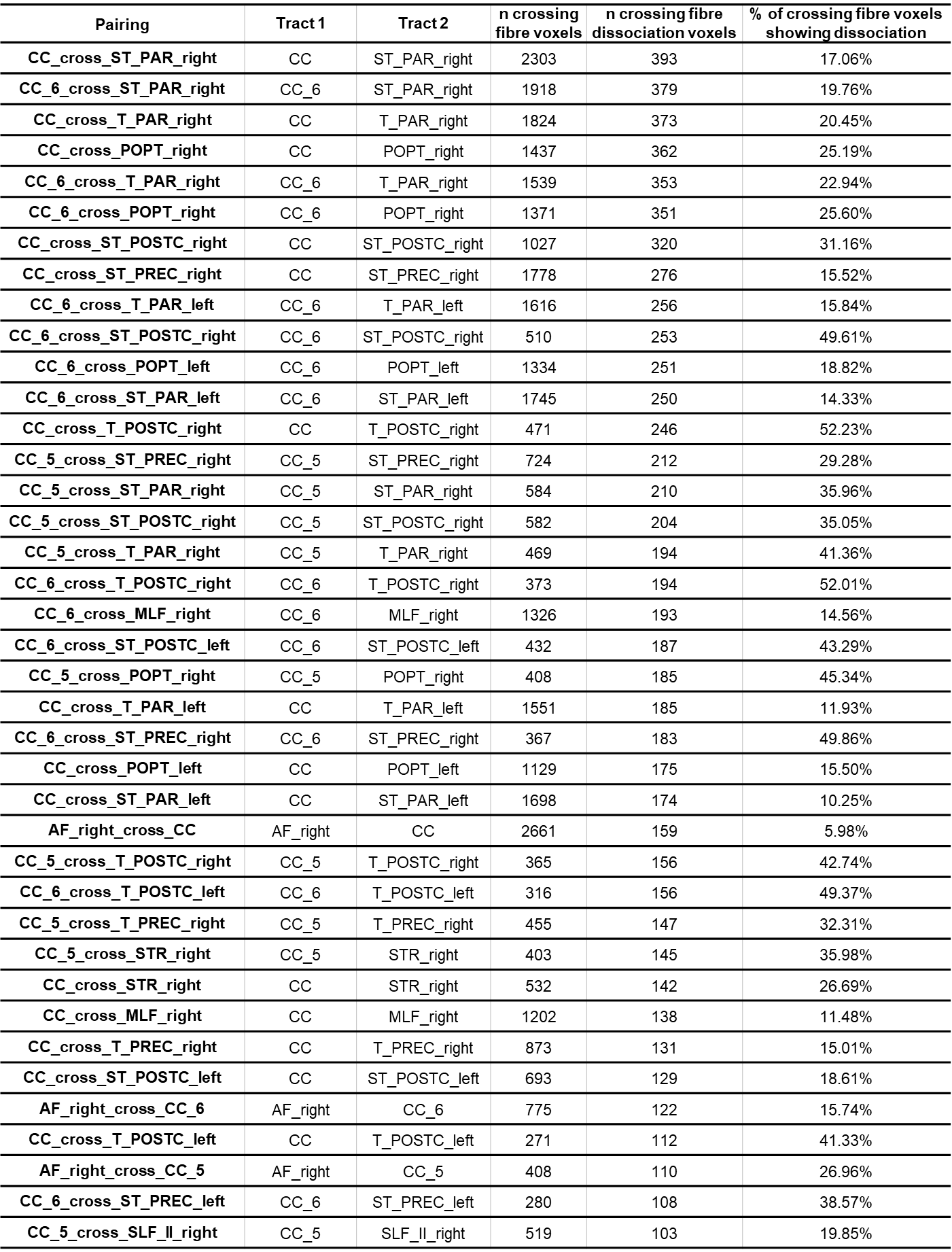


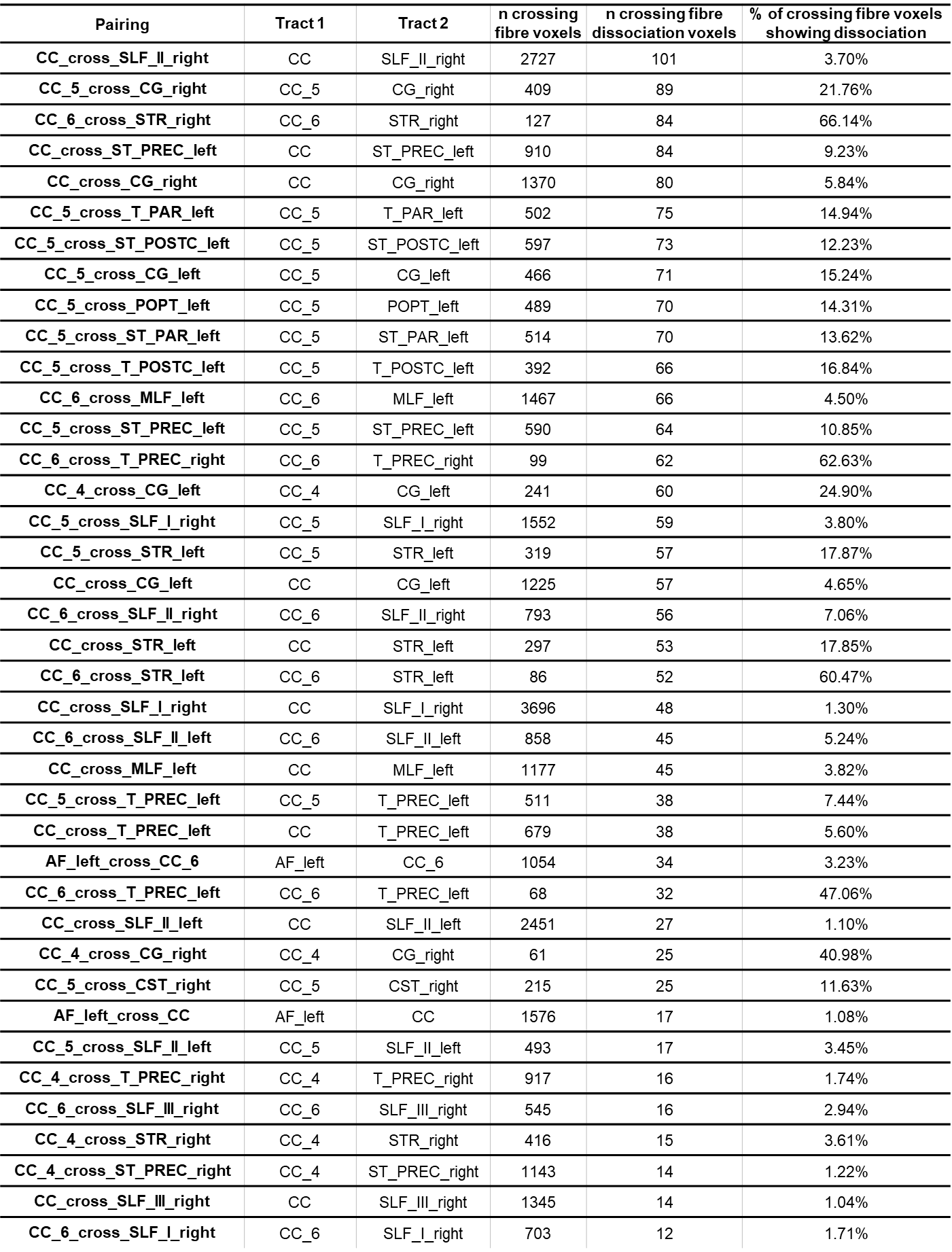


# Supplementary Table 7 – FC filtered crossing fibre tract pairings
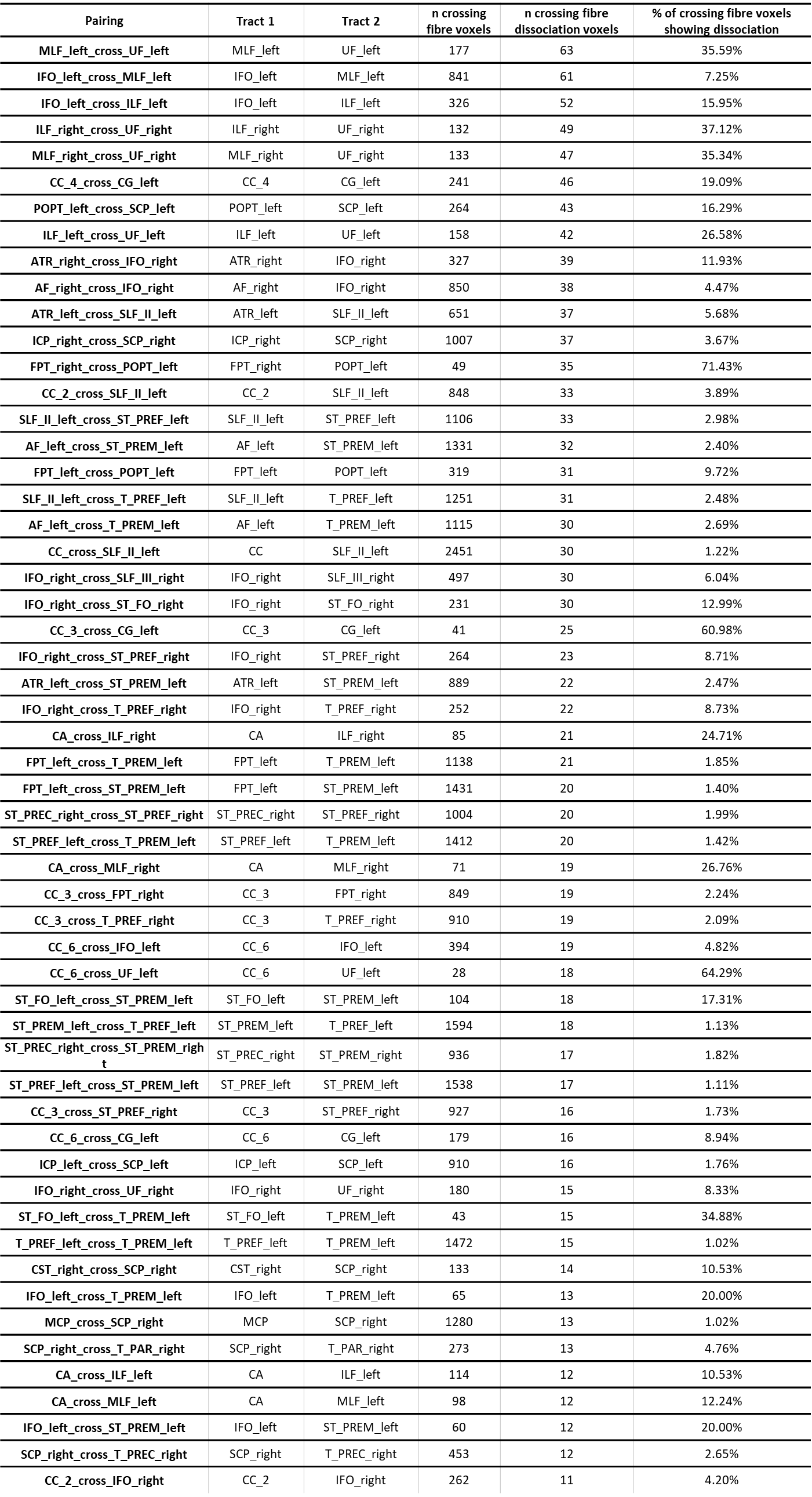


# Supplementary Table 8 – FDC filtered crossing fibre tract pairings


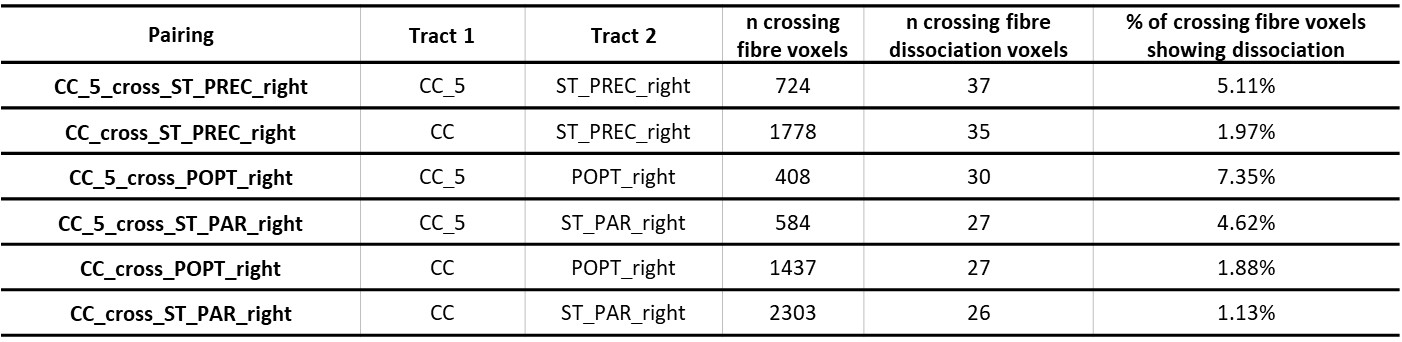


# **Supplementary Figure 2 - Example tracts of interests (TOI) demonstrating differential effects of moderate-severe TBI on white matter structure using FBA.**


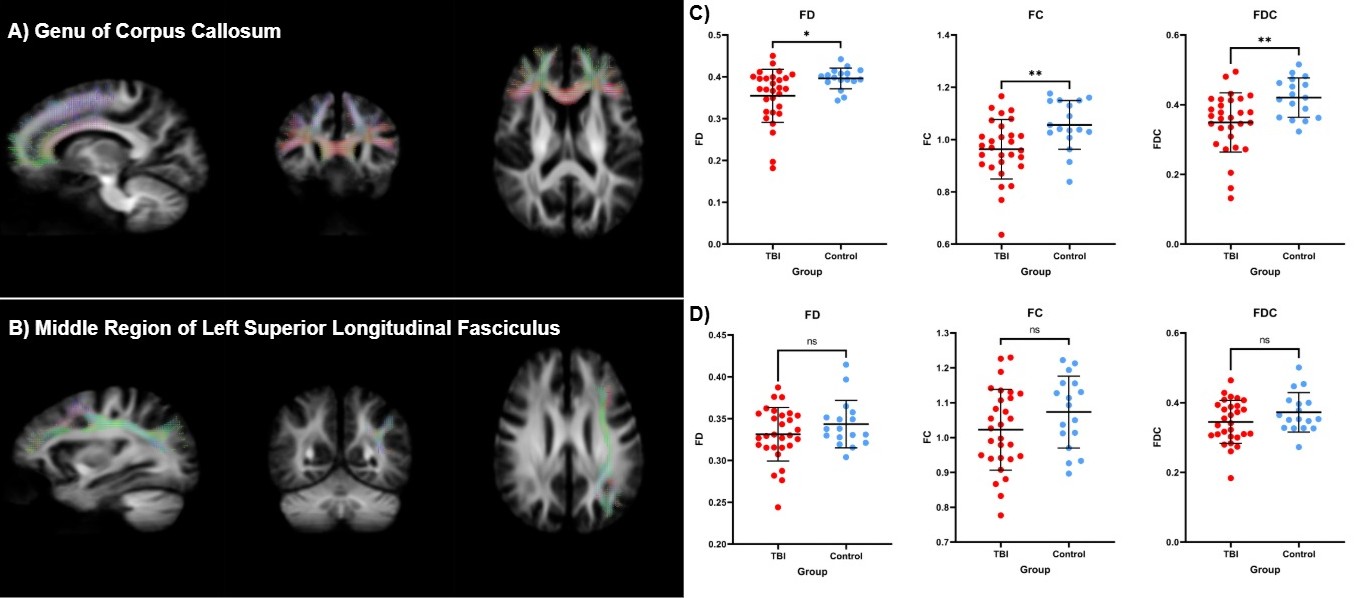
*A and B:* TOI fixel-analysis masks displayed on axial, coronal, and sagittal views of the study-specific fibre orientation distributions (FOD) template, with masked fixels coloured by direction (anterior-posterior: green; superior-inferior: blue; left-right: red). Fixel-analysis masks were produced using Mrtrix3 and TractSeg. *C and D:* Estimation plots representing individual tract-mean fixel-wise metric values for TBI patients (TBI) and healthy controls (Control). The middle line represents group level means and error bars represents one standard deviation. Degree of significance were based on false-discovery rate (FDR) corrected p-values produced from multiple linear regression models adjusted for nuisance covariates (age, sex, and intracranial volume) and robust standard errors.

*ns = non-significant; *P < 0.05; **P < 0.01; ***P < 0.001*

# Supplementary Figure 3 - Large number of tracts demonstrate differential damage in regions of crossing fibre.

1. **FD**

1. **Log FC**
2. **FDC**

*White = Tracts do not cross*

*Green = Tracts cross but do not demonstrate significant areas of differential damage (defined as 1. Number of dissociation voxels > 10; and 2. Dissociation voxels >1% of total crossing fibre voxels)*

*Yellow = Tracts cross and demonstrate significant areas of differential damage, but not differential damage at the whole tract level*

*Red = Tracts cross, demonstrate significant areas of differential damage, and differential damage at the whole tract level*

# Supplementary Figure 4 - Visualisations of correlation between white-matter integrity and delayed recall.


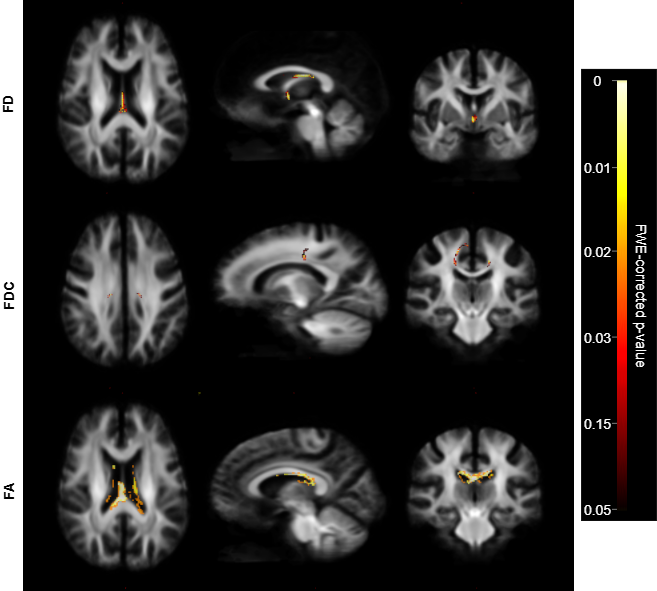


Dual regression analysis was used to identify correlations between measures of white-matter integrity (FBA metrics and voxel-wise FA) and the Hopkins Verbal Learning Test (revised) Delayed Recall across the entire fixel analysis mask in TBI patients, adjusting for nuisance covariates (age, sex, and intracranial volume). Areas thresholded at FWE-corrected *P*-value < 0.05 are displayed across 8 axial slices of the study specific fibre orientation distributions (FOD) template.

# Supplementary Table 9 – Significant correlations between neuropsychological test results with WM-integrity metrics


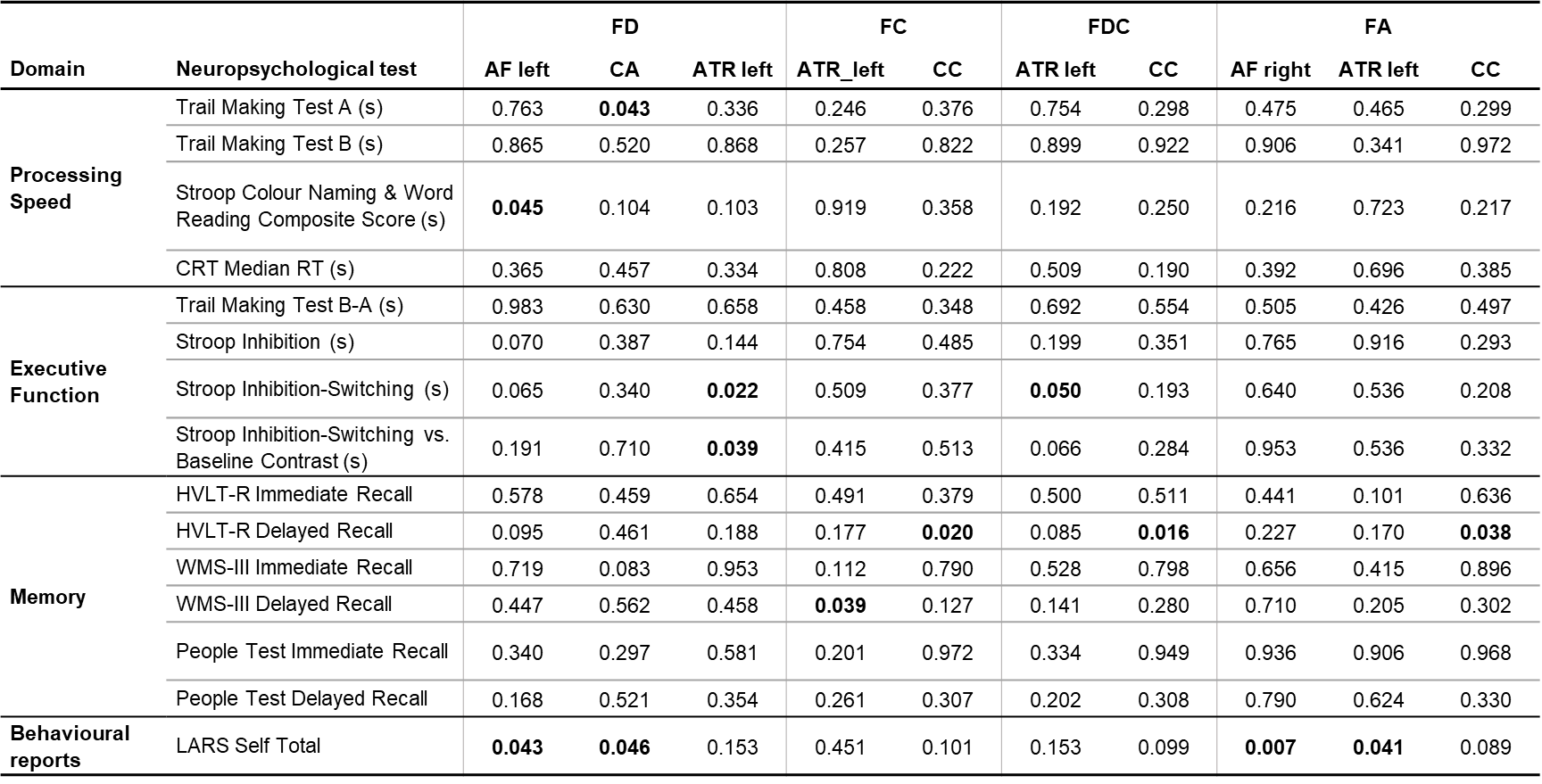


Numbers represent uncorrected p-values. Significant results highlighted in bold. No results survive multiple comparison correction.

Test abbreviations: CRT = Choice reaction time; RT = Reaction time; HVLT-R = Hopkins Verbal Learning Test – revised; WMS-III = Wechsler Memory Scale; WASI = Wechsler Abbreviated Scale for Intelligence; WTAR = Wechsler Test for Adult Reading; LARS = Lille Apathy Rating Scale;

The following list shows the full names of each tract: Arcuate fascicle (AF), Anterior thalamic radiation (ATR), Commissure anterior (CA), Corpus callosum (CC)

**Supplementary References**

1. Malec JF, Brown AW, Leibson CL, Flaada JT, Mandrekar JN, Diehl NN, et al. The Mayo Classification System for Traumatic Brain Injury Severity. *Journal of Neurotrauma*. 2007;24(9): 1417–1424. https://doi.org/10.1089/neu.2006.0245.

2. Benjamini Y, Krieger AM, Yekutieli D. Adaptive linear step-up procedures that control the false discovery rate. *Biometrika*. 2006;93(3): 491–507. https://doi.org/10.1093/biomet/93.3.491.
